# Image-Based Classification of Concrete Carbonation Using YOLO Models

**DOI:** 10.3390/ma19112198

**Published:** 2026-05-23

**Authors:** Yaren Aydın, Ümit Işıkdağ, Sinan Melih Nigdeli, Gebrail Bekdaş, Celal Cakiroglu

**Affiliations:** 1 Department of Civil Engineering, Istanbul University-Cerrahpaşa, 34320 Istanbul, Türkiye; yaren.aydin@iuc.edu.tr (Y.A.); bekdas@iuc.edu.tr (G.B.); 2 Department of Architecture, Mimar Sinan Fine Arts University, 34427 Istanbul, Türkiye; umit.isikdag@msgsu.edu.tr; 3 GameAbove College of Engineering and Technology, Eastern Michigan University, Ypsilanti, MI 48197, USA

**Keywords:** YOLO, classification, carbonation, deep learning

## Abstract

Detecting the presence of carbonation is critical for monitoring structural safety and durability. Identifying the presence of carbonation reveals the risk of chemical changes within the concrete and the potential for reinforcement corrosion. This detection allows for a reliable and prioritized assessment of the structure’s current condition. Therefore, checking for the presence or absence of carbonation is a critical indicator in determining structural safety and maintenance priorities. This study explicitly addresses a critical gap in the literature, where existing carbonation research predominantly focuses on regression-based estimation of carbonation depth, while the problem of direct visual classification of carbonation presence for rapid decision-making currently remains underexplored. In this context, the study aims to fill this research gap through developing a robust and field-applicable deep learning-based classification framework for the automated detection of carbonation presence on concrete surfaces using images, while systematically comparing the performance of different YOLO architectures and assessing the suitability of a previously unused dataset (ConcreteCARB) for carbonation classification tasks. In this context, YOLOv8m, YOLOv11m, YOLOv12m, and YOLOv26m were compared for concrete carbonation classification, aiming to find the most suitable model. The results show that YOLOv8m and YOLOv11m achieve perfect accuracy (Accuracy = 0.9981, Precision = 1, Recall = 0.9964, Specificity = 1, AUC-ROC = 1). In inference efficiency analyses, the YOLOv11m model was identified as the fastest model with the lowest latency and highest FPS. While YOLOv8m and YOLOv26m offered balanced speed-performance results, YOLOv12m showed a relatively lower processing speed. The findings indicate that YOLOv11m is the most suitable option for real-time applications.

## 1. Introduction

Concrete is a basic building material, and its behavior is determined not only by the sum of the properties of its components but also by the interactions between these components [1]. The durability of concrete depends on the correct selection of materials used in the mix and the proper application of the production, pouring, compaction, and curing processes of the concrete. However, providing these conditions alone is not sufficient for a long service life of concrete; concrete must also be protected against environmental effects.

In evaluating existing reinforced concrete structures, concrete compressive strength is a fundamental criterion. Destructive testing involves drilling into the concrete to obtain core samples from the structure. The common core drilling method weakens the cross-section, reduces load-bearing capacity, and leads to time and cost losses [2]. Therefore, determining concrete compressive strength using non-destructive testing methods has gained importance [3]. There are non-destructive methods such as Schmidt surface hardness [4], Ultrasonic Pulse Velocity (UPV), Rebound Hammer (RH), Sonic Rebound (SonReb) [5] and Water absorption [6]. Ultrasonic testing provides information about strength based on the elastic properties of the material by measuring the wave propagation speed within the concrete. The rebound index, on the other hand, measures surface hardness with a sclerometer and is empirically correlated with concrete strength. However, it is highly sensitive to factors such as surface moisture, carbonation, roughness, and concrete age. The SonReb method combines these two non-destructive tests to provide a more reliable strength estimation because the errors of one method are partially offset by the other [7].

**Figure 1:**
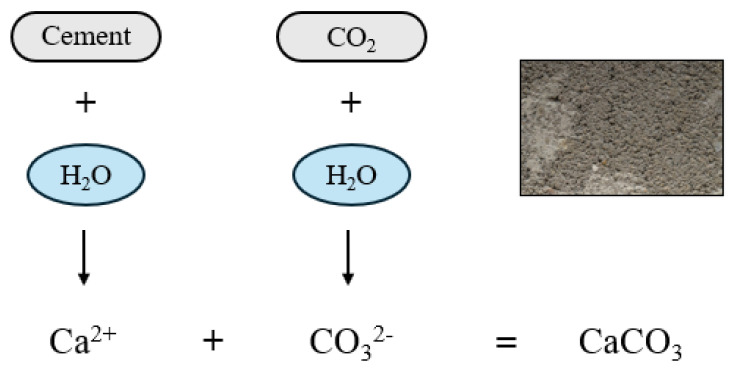
Carbonation reaction.

Many factors affect the durability of concrete, and carbonation is one of them. Carbonation in concrete is the process by which carbon dioxide (CO_2_) gas penetrates the concrete as a result of environmental effects to which the concrete structure is exposed throughout its lifespan, reacting with calcium hydroxide (Ca(OH)_2_) to form calcium carbonate (CaCO_3_). Due to carbon dioxide (CO_2_) in the air, carbonation can occur in all materials containing cement (Figure 1) [8]. The most significant effect of carbonation is lowering the pH of the concrete, thereby increasing the risk of corrosion in the reinforcing steel. Carbonation depends on many factors, including concrete permeability, relative humidity, concrete grade, cover thickness, and time [9]. Non-destructive testing methods used to determine carbonation in concrete are of great importance in evaluating the service life of structures and planning maintenance and repair strategies.

Carbonation occurs when CO_2_ in the atmosphere enters the porous structure of concrete and reacts with calcium hydroxide (Ca(OH)_2_) to form calcium carbonate (CaCO_3_). This process begins with CO_2_ reacting with water in the pores to form carbonic acid (*H*_2_*CO*_3_). Carbonic acid reacts with *Ca(OH)*_2_ to form *CaCO*_3_ and *H*_2_*O*. The process is expressed by the chemical reactions in Equations (1) and (2) [10].

(1) $${CO}_{2}+H_{2}O\to H_{2}$$

(2) $${H_{2}CO}_{3}+Ca({OH)}_{2}\to {CaCO}_{3}+{2H}_{2}$$

Carbonation tests can be performed in two ways: in a natural environment and in a laboratory environment. Experiments in a natural environment are conducted to examine the effect of environmental conditions on carbonation in structures. However, since this process takes many years, accelerated carbonation tests are mostly preferred in practice [11]. Non-destructive testing methods used to determine carbonation in concrete are of great importance in evaluating the service life of structures and planning maintenance and repair strategies.

Carbonation depth is usually determined using a phenolphthalein indicator. Phenolphthalein (C_2O_H_14_O_4_) is a phthalate dye first synthesized by Adolf von Baeyer in 1871. It is a common acid-base indicator due to its colorless appearance in acidic environments and its pink color in basic environments. The color change occurs because its molecular structure changes in basic environments [12]. Phenolphthalein reacts with the concrete surface, causing a pink color to form in areas where carbonation does not occur. For this purpose, the concrete surface is cut and cleaned, and a phenolphthalein solution is applied to the cut surface. Uncarbonated areas turn purple-pink, while no color change is observed in carbonated areas. The depth of carbonation is determined by measuring the colorless areas. Measurements are usually taken at different points of the cut using calipers, and the average value is calculated [13].

The carbonation test in a laboratory environment can be accelerated without the need for years of waiting. In this method, CO_2_ gas or carbonated water is applied to the concrete to provide a higher CO_2_ concentration than atmospheric conditions, accelerating the carbonation process [14].

Carbonation is a process that negatively affects the chemical structure of concrete. It lowers the pH of the concrete, causing corrosion in the reinforcing steel. Factors influencing carbonation include concrete components (aggregate, water, cement, mineral and chemical additives), the pore structure of the concrete, moisture content, insulation properties, concrete class, and cover thickness [15]. If carbonation in concrete reaches very advanced levels, it reduces the structural integrity of the building.

In particular, carbonated and non-carbonated areas can be distinguished by color differences in experimental images obtained using phenolphthalein indicators. However, manual evaluation of such images is time-consuming and can lead to user-related errors.

Therefore, image processing and deep learning (DL) methods offer a significant alternative in carbonation analysis. Deep learning methods automatically analyze color, texture, and shape characteristics in images obtained from concrete samples, enabling the detection of carbonated areas and faster and more objective determination of carbonation depth. This approach both speeds up the measurement process and reduces human-induced evaluation errors. Therefore, image processing-supported machine learning methods stand out as an effective tool for monitoring the durability of reinforced concrete structures and more reliably evaluating carbonation damage.

Accurate, rapid, and repeatable detection of carbonation is of great importance for building safety. Therefore, traditional human-eye-based inspection mechanisms are increasingly being replaced by faster, more objective, and automated systems. With the development of deep learning (DL) techniques, various neural network structures have also become widely used in civil engineering. Modern image processing ANN architectures such as ResNet, Inception, DenseNet, MobileNet, and EfficientNet are used in civil engineering due to their deep, lightweight, or fast structures. These architectures are effectively used in civil engineering for tasks such as the identification of the surface cracks of concrete [16], real-time displacement prediction in nonlinear seismic response of tall buildings [17], concrete gravity dam deformation [18], and detecting cracks in concrete surfaces [19].

Among object detection algorithms, the You Only Look Once (YOLO) family stands out, particularly for its real-time performance. By analyzing the image in a single step, it can quickly determine both the class and location of the object. In addition, YOLO can also be successfully used for classification tasks. Although the YOLO architecture was primarily developed for object detection, its adapted versions for classification tasks have also offered high performance in recent years [20]. In this study, YOLO models were preferred for classifying concrete carbonation from images due to their strong feature extraction capabilities, fast extraction time, and suitability for real-time applications. In addition, the literature has shown that YOLO-based approaches yield successful results in problems such as image-based damage detection [21], identification of concrete pathologies [22] and surface crack detection [23].

### 1.1. Literature Review

In recent years, image-based deep learning methods for detection and classification have been widely used in civil engineering. Dorafshan et al. (2018) [24] compared classical edge detection methods with DCNN-based approaches for image-based crack detection in concrete structures. The AlexNet-based DCNN demonstrated labeling accuracy up to 99%, achieved 86% success in transfer learning, and was able to detect finer cracks. Liu et al. (2023) [25] proposed “R-YOLOv5” by developing the YOLOv5 model for detecting overlapping and directional cracks with large aspect ratios. Trained on 1628 images with 300 iterations, the model achieved 94.03% mAP@0.5 on the test set, outperforming YOLOv5 and other rotational object detection methods. Sohaib et al. (2024) [26] comparatively analyzed different YOLO models on a dataset containing both cracked and uncracked images to detect cracks in concrete structures. The results showed that YOLOv10 performed best with an extraction time of 19.5 ms and a 74.52% mAP. Zhou et al. (2024) [27] used YOLO-v5 to detect surface damage in previously damaged concrete beams, and the results were compared with experimental data. Deviations ranging from −3.03% to 8.66% were observed between the YOLO-v5 results and the experimental data. Raushan et al. (2025) [21] created a dataset consisting of 3750 real images and labels containing different textures, colors, and architectural elements (doors, windows, etc.) and performed damage detection in concrete structures with multi-feature backgrounds. The results showed that YOLO models are effective in complex backgrounds. YOLOv4 provided the best performance, achieving 92.2% sensitivity, 86.8% recall, and 88.9% F1 score. Liang and Xu (2025) [28] proposed an intelligent inspection method that automatically evaluates the appearance quality of precast concrete components using an improved YOLO model and multi-source data. The improved YOLO model demonstrated high performance, achieving an average accuracy of 85.02% in the VOC2007 dataset. Successful detection was achieved for four different defect types, and an accuracy of 0.1 mm was obtained in point cloud-based measurements. These studies generally focus on the detection and segmentation of damage on concrete surfaces, and high accuracy rates have been reported using CNN and YOLO-based methods. However, the visual classification of damage types related to chemical degradation processes such as carbonation, has been addressed more limitedly in the literature. Although carbonation is a mechanism that affects the durability of concrete in the long term, most studies do not consider this type of damage as a direct image-based classification problem. In this context, this study addresses a little-studied topic by focusing on the image-based classification of carbonation damage, as distinct from crack detection.

This section of the study reviews research that uses deep learning for carbonization in general and evaluates the potential of deep learning methods in this field. The studies explained here have mostly been conducted in the last 5 years.

Lee et al. (2020) [29] evaluated a machine learning approach to predict the carbonation behavior of concrete. They made carbonation predictions using a Deep Neural Network model, and the results were compared with the AIJ model and the finite element method (FEM). The differences between the experimental data and the deep learning results were found to be quite small, and higher accuracy was achieved, especially compared to the AIJ model. Giulietti et al. (2021) [30] used image processing and Convolutional Neural Networks to determine the carbonation depth in phenolphthalein-sprayed concrete, separating carbonated and non-carbonated regions and preventing aggregates at the carbonation front from causing measurement errors. A very strong correlation (R^2^ = 0.96) was obtained between the proposed method and the EN 13295 standard. Chen et al. (2022) [31] presented a hybrid machine learning approach combining artificial neural network (ANN) and support vector machine (SVM) models to predict concrete carbonation depth. They created a database containing 532 records and predicted the carbonation depth with high accuracy (r = 0.9788–0.9946) using the ML model. However, hybrid models showed higher correlation and lower error values compared to single ANN and SVM models. Tran et al. (2023) [32] compared the performance of 4 single (XGB, GB, RF, SVM) and 4 hybrid machine learning models (XGB_RRHC, GB_RRHC, RF_RRHC, SVM_RRHC) for predicting the carbonation depth of concrete containing fly ash using 688 samples and 7 input variables. The highest performance was observed in the XGB model with default hyperparameters (R^2^ = 0.9770, RMSE = 2.2725, MAE = 1.5218). Ehsani et al. (2024) [33] predicted carbonation depth on a 37-variable dataset using ANN, random forests, decision trees, and SVM. They used the MOEA/D-ANN (Multi-Objective Evolutionary Algorithm Based on Decomposition and Artificial Neural Networks) method to select the most suitable variables, and compared to the traditional RReliefF method, MOEA/D-ANN yielded the most accurate results. Ma et al. (2025) [34] trained, tested, and compared six machine learning models (KNN, BP-ANN, DT, RF, GB, XGB) for carbonation percentage estimation on 100 carbonated recycled concrete fine aggregate mixtures. Ultimately, the XGB model demonstrated the best performance on the test set, with the highest accuracy (R^2^ = 0.974) and the lowest error (RMSE = 1.521). Liu et al. (2026) [35] used a dataset of 2163 samples obtained from natural and accelerated carbonation experiments and estimated the carbonation depth using Random Forest (RF), two baseline deep learning models (Artificial Neural Network (ANN), Convolutional Neural Networks (CNN)), and two enhanced models with feature interactions, carbonation equations, and attention mechanisms (ATT-ANN, ATT-CNN). The best performance was obtained with the ATT-CNN model (R^2^ = 0.9142), and the MSE value was significantly reduced compared to the RF and CNN models. Jafari and Dorafshan (2026) [36] proposed a full-field optical method based on hyperspectral imaging to determine concrete surface carbonation non-contactly and rapidly. They analyzed the reflectance data of 12 samples with different carbonation levels and performed a total of 200 hyperspectral imaging scans. They validated the carbonation process with X-ray diffraction (XRD), scanning electron microscopy (SEM), and RGB images. They used the K-means clustering method to differentiate between carbonated and non-carbonated regions and achieved 90% IoU accuracy.

This literature review reveals that carbonation studies largely focus on determining the depth of carbonation. In contrast, studies aimed at detecting carbonation using images are very limited.

### 1.2. Research Significance

The construction industry faces multifaceted challenges such as increasing building density and workforce limitations. These challenges have made it imperative to support structural inspection and maintenance processes with digital technologies. In this context, AI-based object detection and image analysis systems offer a significant solution area for time-sensitive and environmentally complex tasks such as the detection of carbonation on concrete surfaces. Early detection of carbonation is difficult and prone to error with classical visual inspection and manual measurement methods. A high-accuracy detection system provides rapid information on the presence/absence of carbonation in concrete elements, enabling engineers and maintenance teams to make quick decisions, which provides operational and cost advantages.

While existing studies generally evaluate using a single model, this study offers a comparative perspective by analyzing different YOLO architectures under the same dataset and experimental conditions. Furthermore, it differs from other studies in the literature by focusing on the visual classification of concrete carbonation. In this respect, the study both provides a contribution specific to the application field and allows for a more objective evaluation of model performance.

**Figure 2:**
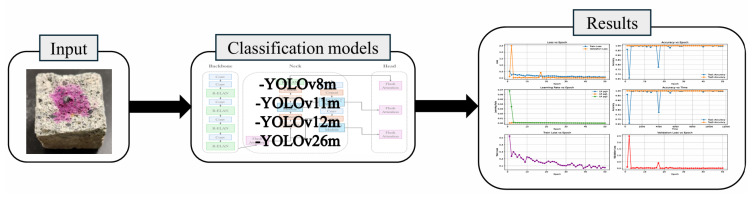
The Overview of the Study.

This study explicitly addresses a critical gap in the literature, where existing carbonation research predominantly focuses on regression-based estimation of carbonation depth, while the problem of direct visual classification of carbonation presence for rapid decision-making currently remains underexplored. In this context, the current study aims to fill this research gap through developing a robust and field-applicable deep learning-based classification framework for the automated detection of carbonation presence on concrete surfaces using images, while systematically comparing the performance of different YOLO architectures (which are known as robust and accurate image classifiers) under consistent experimental conditions. Specifically, the objectives of the study were: (i) to evaluate the capability of multiple state-of-the-art YOLO variants ranging from the best recent CNN backbones to novel architectures that include attention (like) modules (YOLOv8m, YOLOv11m, YOLOv12m, and YOLOv26m) in accurately distinguishing carbonated and non-carbonated concrete surfaces, (ii) to analyze the trade-offs between accuracy, computational efficiency, and model complexity for practical deployment scenarios, and (iii) to assess the suitability of a previously unused dataset (ConcreteCARB) for carbonation classification tasks in civil engineering applications. Methodologically, the study employs a supervised deep learning framework using 903 labeled images obtained via phenolphthalein-based carbonation testing, where carbonation presence is visually encoded through color differences. The dataset is partitioned using a validation strategy with a 80/20% training-validation split, and all models are trained under identical hyperparameter settings to ensure fair comparison. Model performance is evaluated using quantitative metrics including accuracy and confusion matrix-derived indicators, enabling both overall and error-type analysis. The experimental pipeline is implemented using the Ultralytics YOLO framework (Figure 2) within a controlled hardware environment, and comparative analysis is conducted to identify the optimal model configuration that balances predictive performance with computational efficiency for real-world structural inspection applications.

These findings will be beneficial for field applications, particularly providing opportunities for optimized solutions that can be used in unmanned inspection vehicles, for automatic building inspections and intelligent maintenance systems.

### 1.3. Novelty and Contributions

This study proposes a practical deep learning-based framework for automated carbonation detection in concrete using image data. In contrast to the majority of existing studies that focus on regression-based estimation of carbonation depth, the problem is reformulated as a binary classification task targeting rapid identification of carbonation presence, which is more aligned with real-world inspection and decision-making processes.

The main contributions of this study are as follows:

The study introduces a classification-oriented perspective to carbonation assessment, enabling faster and more practical evaluation compared to traditional depth estimation approaches.

A systematic and controlled evaluation of recent YOLO variants is conducted, providing clear insights into their relative performance and suitability for carbonation detection under consistent conditions.

The results demonstrate that high accuracy can be achieved alongside computational efficiency, highlighting the potential of these models for real-time and resource-constrained structural inspection applications.

Overall, this study contributes by establishing an application-driven framework that aligns modern deep learning capabilities with the practical requirements of carbonation detection, supported by a consistent and comparative experimental analysis.

## 2. Materials and Methods

This section will present the image dataset used in the study, the architecture of the YOLO models, and the performance evaluation metrics used in this study. Technical information about the hardware infrastructure on which the training is done will also be presented.

### 2.1. Dataset

In this study, the publicly available ConcreteCARB dataset [37] was used to examine the presence/absence of carbonation in laboratory-prepared concrete samples. This dataset contains 903 high-resolution concrete images tested for carbonation using the phenolphthalein indicator method. The images are classified into two categories based on their carbonation status: 431 images are Carbonated concrete samples, and 472 images are Non-carbonated concrete samples. The dataset was balanced class-wise. Each image shows the surface of a crushed concrete prism. Carbonated areas are colorless; non-carbonated areas are pink. Samples were prepared with different water-cement ratios and additives (industrial silica, Opuntia ficus-indica extract) and photographed with a Samsung SM-S901U1 smartphone (4000 × 3000, no flash) after 180 days of natural exposure [37]. All 903 images were labeled by expert engineers as indicated by Guzmán-Torres [37]. The labeled dataset was divided into training and validation sets with a 4:1 ratio, consisting of a total of 903 labeled samples. Although the original images were high-resolution, all experiments were performed with a 640 × 640 input size. This allowed the model to effectively learn task-oriented features and exhibit strong generalization performance by preserving critical color contrast and carbonation regions.

To ensure the reliability and reproducibility of the high accuracy values reported in the study, the dataset was divided into 80% training and 20% testing, and all experiments were repeated with 5-fold cross-validation. Three different seed values (42, 123, and 456) were used to evaluate the model’s sensitivity to randomness.

ConcreteCARB dataset was obtained under controlled laboratory conditions. Images were recorded from different shooting distances and angles using mobile phone cameras. This resulted in limited variability in lighting and perspective in the dataset. The dataset only includes surface-level carbonation and has a binary classification structure of Carbonated and Non-Carbonated. Partial or intermediate carbonation cases were excluded. As stated by the dataset providers [37], this structure created in a laboratory environment offers a more controlled and homogeneous distribution compared to concrete surfaces in real field conditions. Therefore, the impact of environmental factors (light variations, external environmental effects, and surface heterogeneity) on model performance remains limited. Despite all these limitations, the dataset offers a fundamental area of study for carbonation detection and classification tasks in civil engineering applications and allows for the evaluation of model performance under controlled conditions.

The training set is the main dataset used in the learning process of the deep learning model and is also used for improving the model’s generalization ability. The model learns input-output relationships and recognizes patterns within the data using a training dataset. The validation set, on the other hand, is used for hyperparameter adjustments during training. The model hyperparameters are adjusted according to their performance on this set, preventing overfitting.

**Figure 3:**
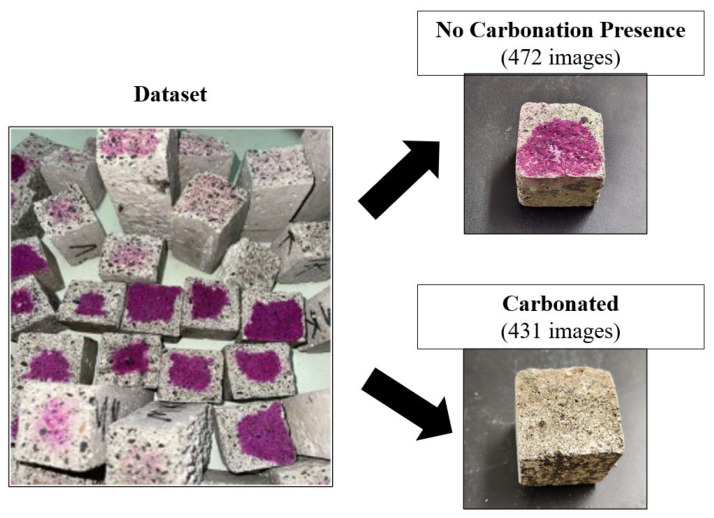
Dataset structure and class distribution.

The structure and class distribution of the dataset are visually presented in Figure 3.

### 2.2. Deep Learning (DL)

**Figure 4:**
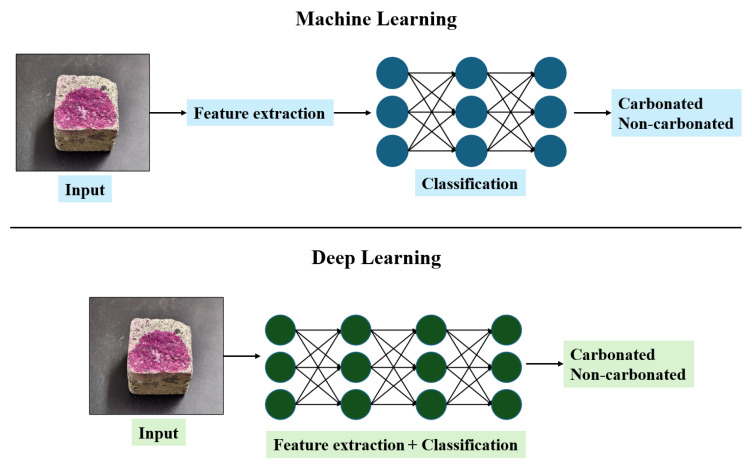
Comparison of machine learning and deep learning [42].

Artificial Intelligence (AI) is an interdisciplinary field that aims to teach learning, decision-making, and problem-solving skills by mimicking human intelligence. It has sub-fields such as machine learning, deep learning, and Natural Language Processing (NLP). Deep Learning (DL) is one of the fastest-growing and most influential subfields of Artificial Intelligence (AI). DL is a Machine Learning (ML) approach that can learn abstract and multi-level representations of data using multilayer artificial neural networks [38]. One of the advantages of DL models is their ability to self-reveal complex relationships in data. For example, in an image processing problem, it transforms low-level edge information into high-level object or conceptual representations [39]. Deep learning models can automatically extract meaningful features from raw data and analyze a wide variety of data types [40]. By identifying complex patterns in large datasets, they contribute to decision support processes [41]. Figure 4 shows the comparison of machine learning and deep learning with feature extraction.

Training DL models requires high computational power, large datasets, and optimized algorithms. Hardware accelerators like GPUs and TPUs enable practical applications [43]. Deep learning is used in civil engineering in areas such as crack detection [44], structural health monitoring [45], detection of personal protective equipment (PPE) at construction sites [46] and other areas of civil engineering [47]. In this study, You Only Look Once (YOLO), one of the DL models, was used for image-based carbonation classification.

### 2.3. You Only Look Once (YOLO)

**Figure 5:**
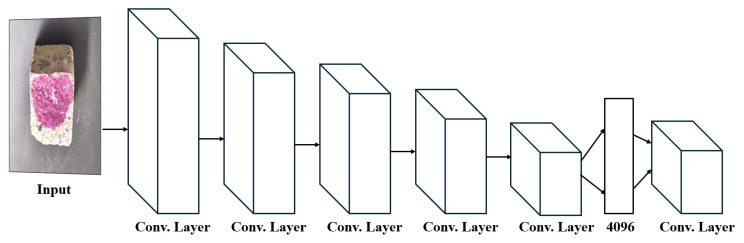
YOLO architecture [48].

You Only Look Once (YOLO) is a deep learning algorithm that excels in both speed and accuracy. The YOLO algorithm was developed by Redmon et al. [48] in 2015 for real-time object detection. YOLO can also be used for other image processing tasks such as classification, segmentation and pose estimation. By processing the entire image at once, it detects objects quickly and efficiently and provides high accuracy in natural images by learning contextual information. As its name suggests, it detects objects by processing the image in a single pass. YOLO performs object detection with a single neural network evaluation. The input image is divided into an S × S grid, and each cell determines whether the object center is within it. Each cell generates multiple bounding boxes, confidence scores for these boxes, and class probabilities. This holistic approach allows the algorithm to operate both quickly and with high accuracy [48]. YOLO uses a convolutional neural network (CNN) structure to extract features such as edges, textures, and shapes from an image, and processes this information to determine the location and class of objects. Feature maps are combined to better perceive objects at different scales [49]. Figure 5 shows the YOLO architecture.

YOLO treats object detection as a single regression problem. Unlike traditional two-stage detectors, it processes the image in a single neural network pass, simultaneously estimating both bounding boxes and class probabilities. This approach makes YOLO fast and efficient for real-time applications [48]. The YOLO family has been refined over time in terms of accuracy and speed. With its new backbone architectures, advanced loss functions, and anchor-free detection methods, it is widely used today.

In this study, the medium dimensions of the most current versions of the YOLO family, YOLOv8, YOLOv11, YOLOv12, and YOLOv26, were used. Table 1 presents the features of the YOLO models used for carbonation classification in this study.

#### 2.3.1. YOLOv8

YOLOv8 was released by Ultralytics [50] in 2023. In addition to object detection, it supports segmentation, exposure estimation, tracking, and classification. Thanks to its anchor-free structure and decoupled head architecture, position, class, and objectivity estimations are learned separately, increasing accuracy [54].

**Figure 6:**
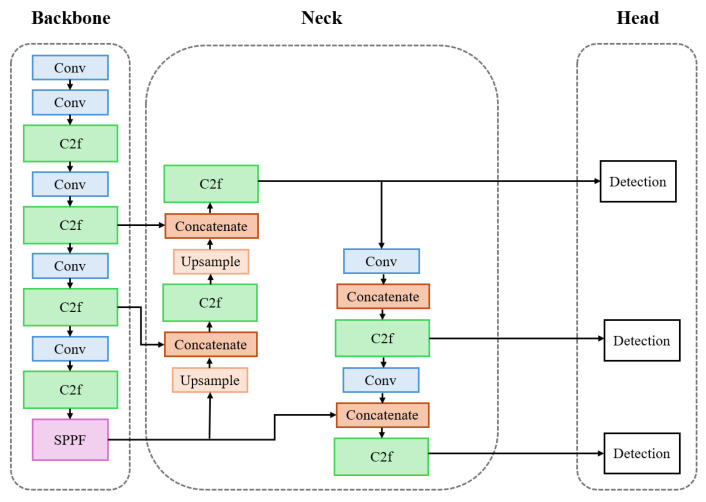
YOLOv8 architecture [55].

The YOLOv8 model consists of three main sections: the backbone, the neck, and the head. Figure 6 shows a visual diagram of this structure.

In YOLOv8, unlike previous versions, the three convolutional layers in the CSP structure have been replaced with the C2f module, which provides more efficient feature learning. This allows the model to learn deeper and more effective representations. The SPPF structure is preserved, combining features at different scales and strengthening spatial context. The neck portion is supported by the C2f module. The model produces output at three different scales for small, medium, and large objects. Furthermore, the classification and regression processes are decoupled, resulting in more efficient prediction [56].

#### 2.3.2. YOLOv11

**Figure 7:**
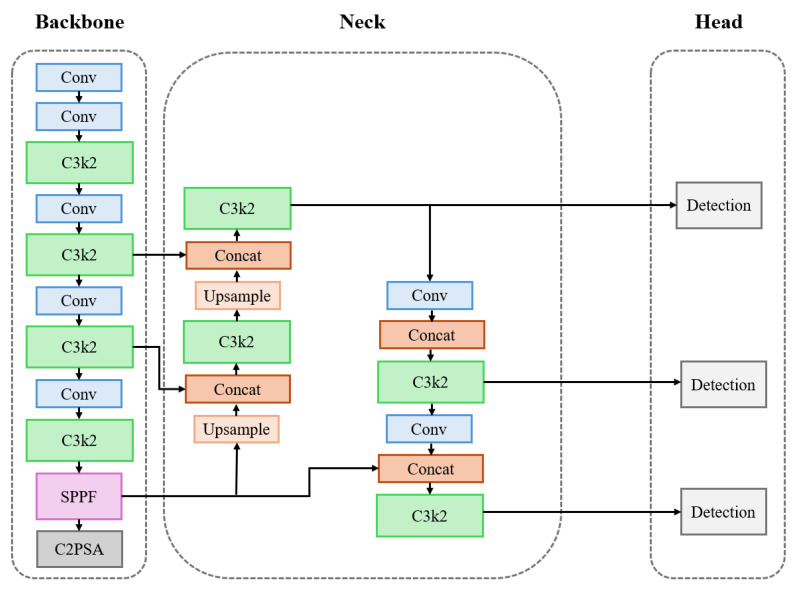
YOLOv11 architecture [57].

YOLOv11 is an optimized version of YOLOv8, designed to deliver higher performance with fewer parameters. The redesigned backbone and neck structures, along with the added C3k2 component, enhance feature extraction capabilities. The C3k2 layer, used instead of the C2f derivative preferred in previous versions of the CSP, aims to increase computational efficiency. This structure uses two small convolutions instead of one large convolution, and k = 2 represents the core size. Thus, performance is maintained while cost reduction and feature extraction are improved. Features are transferred to the head using the C3k2 and C2PSA layers in the neck section. The head structure is designed like the YOLOv8 architecture. The final detection head is formed by assembling C3k2 modules used at different depths and regions [51]. The basic structure of the YOLOv12 architecture is schematically presented in Figure 7.

#### 2.3.3. YOLOv12

**Figure 8:**
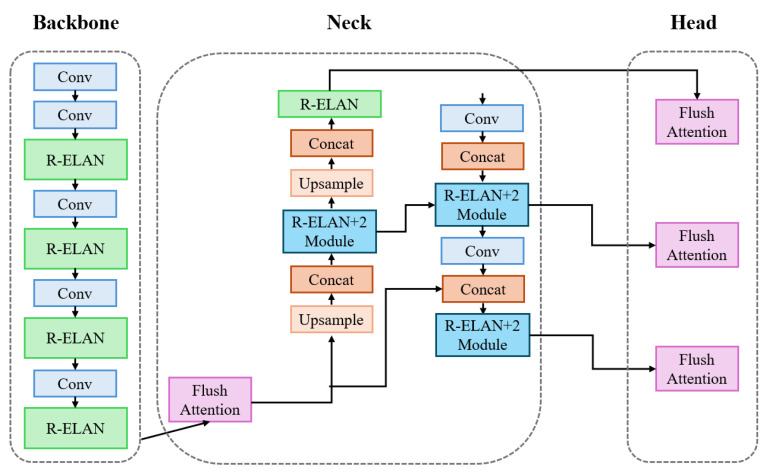
YOLOv12 architecture [59].

YOLOv12 [52] deviates from the CNN-based approach by offering an attention-centric architecture. YOLOv12 accelerates self-attention calculations by utilizing advanced attention mechanisms like Flash Attention in its architecture, enabling efficient operation on large feature maps. Furthermore, thanks to R-ELAN (Residual ELAN) blocks, the training stability of deep networks with residual connections is increased, and the deepening problems seen in YOLOv11 are reduced [58]. This model aims to reduce, or even completely eliminate, the need for CNN-based methods. The main goal of YOLOv12 is to equalize the speed of attention-based models with Convolutional Neural Network (CNN)-based models. In this context, solutions have been provided to the problems of second-order computational complexity and memory inefficiency in attention mechanisms. The Field Attention Module (A2) reduces computational cost while preserving the receptive field, and memory access delays are eliminated through the use of FlashAttention. The A2 module provides high speed and efficiency by segmenting the feature map [52]. Figure 8 shows YOLOv12 architecture.

As shown in Figure 8, the architecture consists of three main sections. First, the backbone, containing R-ELAN blocks, extracts basic and semantic features from the input image. Then, the neck combines and enhances these feature maps, enabling more effective detection of objects of varying sizes. Finally, the head section processes these enhanced features, generating bounding box and class predictions for each object.

#### 2.3.4. YOLOv26

**Figure 9:**
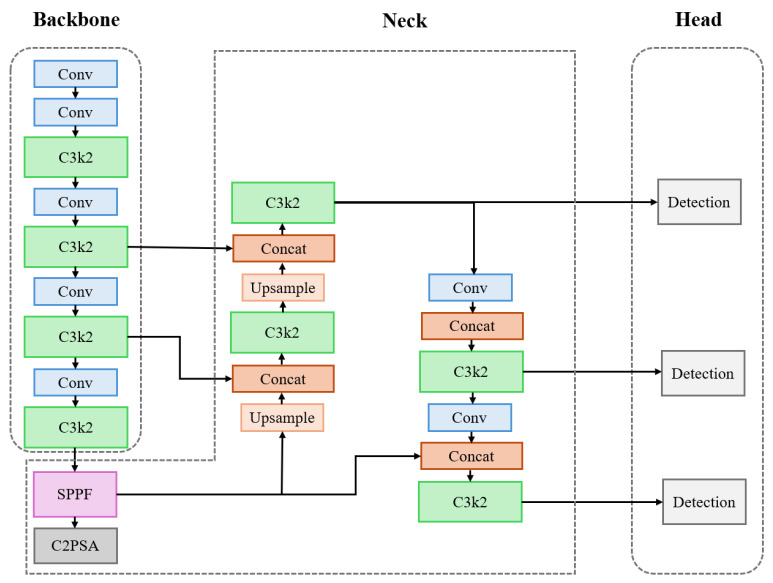
YOLOv26 architecture [60].

YOLOv26 (2025) focuses on deployment rather than complicating the architecture. Innovations include an end-to-end estimator without NMS, DFL removal, and MuSGD optimization. This reduces latency, improves performance on low-power CPUs and edge devices, and supports multi-vision tasks. The YOLOv26 architecture moves away from the trend of increasing parameter complexity and focuses on computational intensity and deterministic latency; this is achieved through pipeline restructuring and optimization strategies such as MuSGD [58]. Figure 9 shows a visual diagram of YOLOv26.

A comparison of the YOLO models used in the study is given in Table 2.

### 2.4. Model Evaluation

Data separation and performance validation are critical steps in evaluating the generalization ability of machine learning and deep learning models. The dataset is typically divided into training and validation sets. In this study, the labeled dataset consisting of a total of 903 samples was divided into 80% training and 20% holdout, and training was conducted with 80% split set using the 5-fold cross-validation method.

This section elaborates on the performance evaluation methods used to objectively and comparably demonstrate the classification performance of the trained deep learning models. Quantitative performance analysis was conducted using common success metrics such as Accuracy, Precision, Recall, F1 score, Specificity, AUC-ROC.

To ensure the reliability and reproducibility of the high accuracy values reported in the study, the dataset was split into 80% training and 20% holdout, and all experiments were repeated with 5-fold cross-validation. Furthermore, the model’s initial weights and sensitivity to randomness in data splitting were analyzed under three different seeds (42, 123, 456).

The models were run separately using three different random initial seed values (seed = 42, 123, 456) to evaluate the consistency and robustness of the results. The results are reported as the average of three independent runs with different random seed values, thereby improving the robustness and reliability of the findings.

#### 2.4.1. Confusion Matrix

A confusion matrix displays the performance of classification models in a tabular format using actual and predicted labels (Table 3). Beyond measuring overall accuracy, it provides qualitative analysis by revealing the type and frequency of errors made by the model.

#### 2.4.2. Accuracy

Accuracy is the most fundamental and frequently used metric in classification problems. It expresses how many of the model’s total predictions are correct. The accuracy formula is given in Equation (3).

(3) $$Accuracy=\frac{TP+TN}{TP+TN+FP+FN}$$

#### 2.4.3. Precision

Precision measures how many of the samples predicted to be positive are actually positive. It evaluates a model’s ability to correctly classify positive or negative labels [61]. Equation (4) is used in the calculation.

(4) $$Precision=\frac{TP}{TP+FP}$$

#### 2.4.4. Recall (Sensitivity or True Positive Rate (TPR))

Recall indicates how many of the true positive samples are correctly captured. It measures how many of the true positive samples were correctly classified [61]. Equation (5) is used in the calculation.

(5) $$Recall=\frac{TP}{TP+FN}$$

#### 2.4.5. F1 Score

The F1 Score is the harmonic mean of Precision and Recall. It provides a single value by balancing the two metrics [61]. Equation (6) is used in the calculation.

(6) $$F1\;Score=2\times \frac{Precision\times Recall}{Precision+Recall}$$

#### 2.4.6. Specificity

Specificity is the number of correctly classified negative samples. Specificity measures how correctly the model classifies genuine negative samples as negative [61]. Specificity formula is given in Equation (7).

(7) $$Specificity=\frac{TN}{TN+FP}$$

#### 2.4.7. AUC-ROC (Area Under Curve–Receiver Operating Characteristic)

The Receiver Operating Characteristic (ROC) curve is a graph calculated for different threshold values. The ROC curve shows the relationship between the True Positive Rate (TPR) and the False Positive Rate (FPR). The area under the ROC curve is expressed as the Area Under Curve (AUC) [62]. A higher AUC value indicates superior model performance.

#### 2.4.8. Other Metrics

To accurately and comprehensively evaluate model performance, the following metrics were also used in this study: Loss measures the difference between the model’s predictions and the actual labels. metrics/accuracy_top1 is the percentage of the model’s most likely prediction that is correct. val/loss is the loss in the validation set. It is used to check for overfitting during training. lr/pg0, lr/pg1, and lr/pg2 are learning rate values applied to different parameter groups. Details regarding these values for the YOLO models are provided in the Supplementary Material to maintain textual integrity.

### 2.5. K-Fold Cross Validation

**Figure 10:**
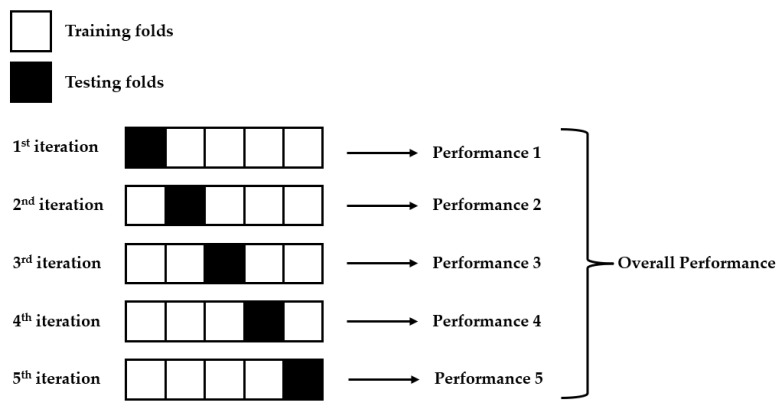
K-fold cross-validation.

K-fold cross-validation was applied to evaluate the model’s generalization ability and reduce overfitting. The aim was to provide a more reliable method for assessing the stability and robustness of models under different data distributions. In k-fold cross-validation, the dataset is divided into k equal parts, and each time one part is used to test the performance of the algorithms, while the remaining k-1 parts of the dataset are used to train the models as shown in Figure 10. In this study, the parameter k was determined as 5.

### 2.6. Experiment Settings

All experiments were performed on Windows 11 Pro using Python 3.10.11 and the Ultralytics YOLO framework (v8.2.18). The computer used in this study features an NVIDIA GeForce RTX 3090 graphics card, an Intel Core i9-13900 processor, 64 GB of RAM and an SSD. The system uses a 64-bit operating system and an x64-based processor architecture. Since YOLO models benefit from GPU acceleration, training processes were performed on the GPU. The obtained training times depend on the hardware conditions used; therefore, the results should be evaluated as relative performance comparisons within the same system. It should be noted that training times may vary with different hardware configurations.

The main hyperparameter configurations used for training (Table 4) were: The epochs value is set to 50. This means the model will be trained on the data for a total of 50 rounds. In YOLO models, imgsz = 640 means that the input images are rescaled to 640 × 640 pixels. A 640 × 640 resolution provides sufficient performance for detecting medium and large-scale objects while keeping training time and GPU usage at reasonable levels. The batch = 64 expression indicates that the model will be updated on small data chunks (batches) of 64 samples in each training step. Batch size is the number of samples to be fed into the network in each iteration.

An early stopping mechanism was used to prevent overfitting during the training of all models. The maximum number of epochs was set to 50, and the patience parameter was set to 10. If no improvement was observed after 10 consecutive epochs, training was automatically terminated. This ensured that training stopped at the point where the model reached its best generalization performance.

Hyperparameter settings used in the experiment (Table 4) aimed to ensure the balance between hardware capacity and the generalization performance of the model [63,64].

## 3. Results

In the experiment, medium-sized models were used for all tested YOLO versions. In this section, the performance metrics of all trained models are presented quantitatively. A comparative table summarizing the overall performance of the models is also included in this section.

In this study, the performance of different YOLO-based models was comprehensively compared (Table 5). Five-fold cross-validation and independent testing revealed that the YOLOv8m and YOLOv11m models exhibited equivalent and the highest performance across all evaluation metrics (accuracy, precision, recall, F1-score, and AUC-ROC). Both models demonstrated stable learning behavior with approximately 0.998 accuracy, 1.00 precision, and 0.998 F1-score values. In contrast, the YOLOv12m and YOLOv26m models showed relatively lower performance, particularly in the recall and F1-score metrics. The performance obtained with YOLOv11m confirms that the analyses yielded consistent results with current architectures.

Although the maximum number of epochs was set at 50, training was terminated earlier depending on the model’s convergence behavior. Specifically, the models converged at different epoch numbers such as 13, 14, and 21 (Supplementary Material); this indicates that the early termination mechanism actively prevents unnecessary training and potential overfitting.

### 3.1. Results for YOLOv8m

**Figure 11:**
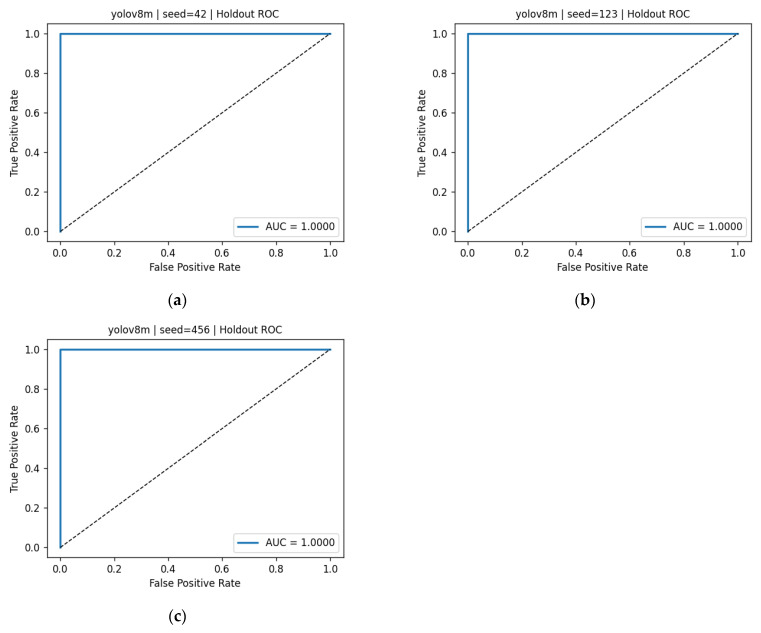
(**a**) ROC curve of the YOLOv8m on the hold-out test dataset (seed = 42); (**b**) ROC curve of the YOLOv8m on the hold-out test dataset (seed = 123); (**c**) ROC curve of the YOLOv8m on the hold-out test dataset (seed = 456).

Figure 11 shows that the ROC curves obtained in the holdout test dataset for three different random seed values (42, 123 and 456) of the YOLOv8m model are quite similar to each other. The positioning of the ROC curves near the upper left corner indicates that the model exhibits high performance in distinguishing between positive and negative classes. Furthermore, the AUC value of 1.00 in all three experiments reveals that the model’s classification success is extremely high and stable. These results demonstrate that the YOLOv8m model offers consistent and reliable prediction performance at different seed values.

Table 6 presents the results obtained separately for each seed value of YOLOv8m. The fact that the performance metrics are quite close to each other among the seeds indicates that the model gives consistent results under different random initial seed conditions and has high stability. The small difference between the seeds indicates that the model is not significantly affected by random variations.

Table 7 shows the mean performance of the results obtained from different baseline values for the YOLOv8m value. High accuracy, precision, recall, F1-score, specificity, and AUC-ROC values, along with a very low standard deviation, indicate that the YOLOv8m model exhibits successful and balanced classification performance on the holdout test dataset. In particular, the AUC-ROC value being close to 1 reveals that the model has a very strong capacity to distinguish between classes.

The YOLOv8m model demonstrated high accuracy and discrimination performance according to aggregated holdout results. Per-seed results revealed that the model exhibited a robust and reliable structure, producing similar outputs across different random seed values. The low standard deviation values support the stability of performance results across seeds.

**Figure 12:**
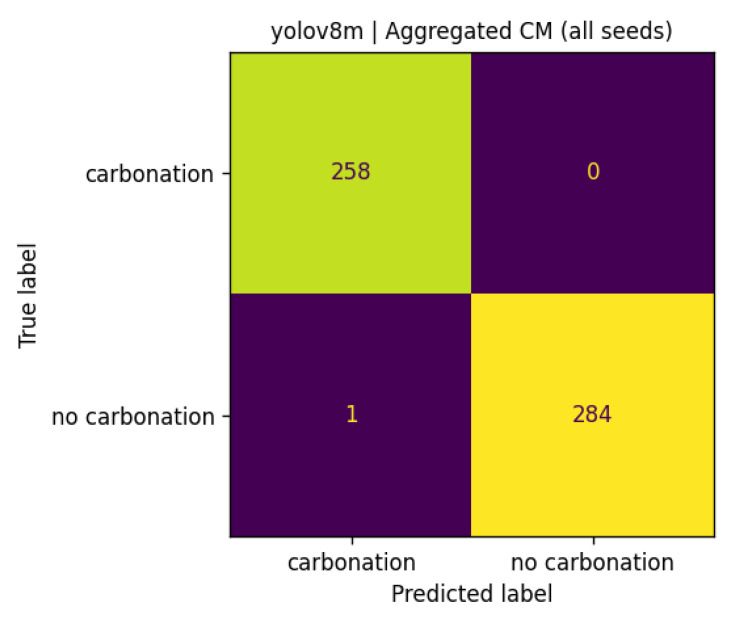
Aggregated confusion matrix obtained from repeated experiments using different random seeds (42, 123, 456) for YOLOv8m.

Figure 12 shows the aggregate confusion matrix obtained from repeated experiments using different random seed values (42, 123, 456) for the YOLOv8m model. According to the matrix values in the two-class classification problem, all 258 samples belonging to the first class were correctly classified, with no misclassification observed. In the second class, 284 samples were correctly predicted, while only 1 sample was misclassified as belonging to the first class.

These results demonstrate that the model has a very high discrimination success rate in both classes. In particular, the extremely low number of false positive and false negative errors reveals that the YOLOv8m model is a stable, reliable, and highly accurate classifier. The fact that only 1 out of 543 samples was incorrectly predicted shows that the model’s performance is quite robust.

#### 3.1.1. Seed42

In this study, the dataset was divided into five equal subsets using a 5-fold cross-validation method. The similarity of metric values across different folds (Table 8) demonstrates that the model exhibits stable and consistent performance across different folds. When the 5-fold cross-validation process and the constant randomness value of seed = 42 are evaluated together, it is seen that the model shows high stability against both data splitting and initial seed conditions.

The close proximity of the training and validation metrics indicates that the model is not overfitting. This suggests that the model can successfully generalize the learned patterns to new data. High Precision and Recall values together indicate that the model keeps both false positives and false negatives at a low level.

Five-fold cross-validation results demonstrate that the YOLOv8m model exhibits high and consistent performance in both training and validation phases. Metrics close to 0.99 and low standard deviations reveal the model’s stability across different folds. Furthermore, the high performance in the holdout set indicates that the model has a strong generalization capacity not only on training data but also on unfamiliar data. Table 9 shows the performance summary of YOLOv8m model (seed = 42) under Cross-Validation and Holdout Settings.

#### 3.1.2. Seed123

The close proximity of the metrics in Table 10 across different folds indicates that the model exhibits stable and consistent performance. The use of 5-fold cross-validation and seed = 123 reveals that the model has high stability against data splitting and randomness conditions. The closeness of the training and validation results indicates that there is no overfitting and that the model generalizes well.

Five-fold cross-validation results demonstrate that the YOLOv8m model exhibits high and consistent performance in both training and validation phases. Metrics close to 0.99 and low standard deviations reveal the model’s stability across different folds. Furthermore, the high performance in the holdout set indicates that the model has a strong generalization capacity not only on training data but also on unfamiliar data. Table 11 shows the performance summary of YOLOv8m model (seed = 123) under Cross-Validation and Holdout Settings.

#### 3.1.3. Seed456

The close proximity of the metrics in Table 12 across different folds indicates that the model exhibits stable and consistent performance. The use of 5-fold cross-validation and seed = 456 reveals that the model has high stability against data splitting and randomness conditions. The closeness of the training and validation results indicates that there is no overfitting and that the model generalizes well.

Five-fold cross-validation results demonstrate that the YOLOv8m model exhibits high and consistent performance in both training and validation phases. Metrics close to 0.99 and low standard deviations reveal the model’s stability across different folds. Furthermore, the high performance in the holdout set indicates that the model has a strong generalization capacity not only on training data but also on unfamiliar data. Table 13 shows the performance summary of YOLOv8m model (seed = 456) under Cross-Validation and Holdout Settings.

### 3.2. Results for YOLOv11m

**Figure 13:**
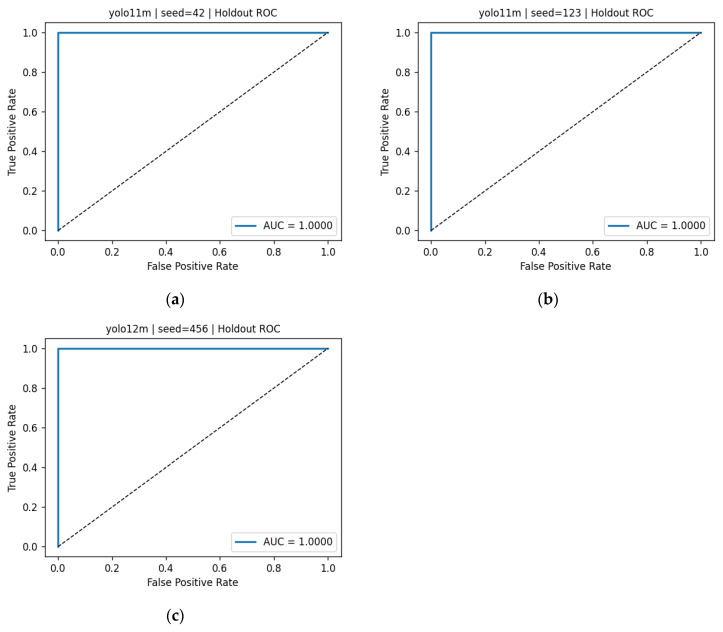
(**a**) ROC curve of the YOLOv11m on the hold-out test dataset (seed = 42); (**b**) ROC curve of the YOLOv11m on the hold-out test dataset (seed = 123); (**c**) ROC curve of the YOLOv11m on the hold-out test dataset (seed = 456).

Figure 13 shows the ROC curves obtained in the holdout test dataset for three different random seed values (42, 123, and 456) of the YOLOv11m model. The ROC curves obtained for seed values of 42, 123, and 456 in the YOLOv11m model show a large degree of overlap. The fact that the curves are close to the upper left corner indicates that the model has a very high class discrimination power. The AUC values being close to 1.00 show that the model’s performance is stable even with different seed values.

Table 14 shows the results obtained for each seed value of the YOLOv11m model. The similarity of the performance metrics indicates that the model provides consistent and stable results under different random seed conditions.

Table 15 shows the mean performance obtained from different seed values for YOLOv11m. High accuracy, sensitivity, recall, F1-score, specificity, and AUC-ROC values, along with low standard deviation, indicate that the model exhibits successful, stable, and balanced classification performance on the test dataset. In particular, the AUC-ROC value being close to 1 reveals that the model has a very high power to distinguish between classes.

**Figure 14:**
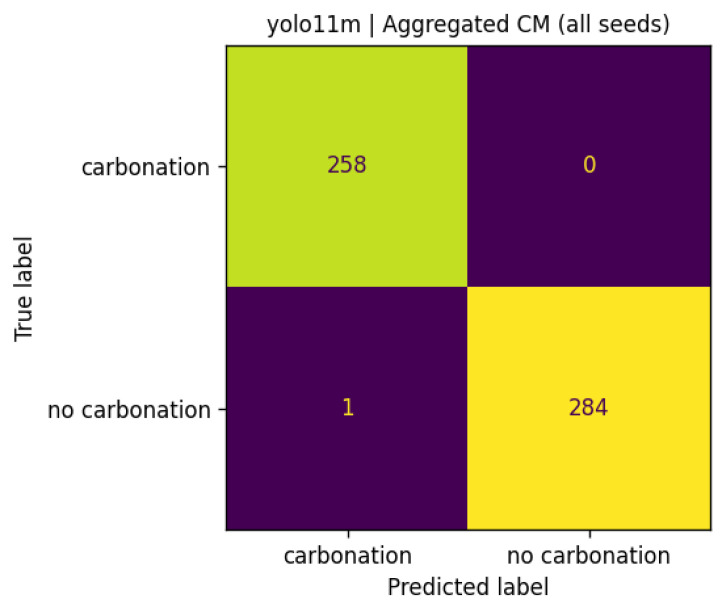
Aggregated confusion matrix obtained from repeated experiments using different random seeds (42, 123, 456) for YOLOv11m.

Figure 14 shows the aggregate confusion matrix obtained from repeated experiments using different random seed values (42, 123, 456) for the YOLOv11m model. According to the matrix values in the two-class classification problem, all 258 samples belonging to the first class were correctly classified, with no misclassification observed. In the second class, 284 samples were correctly predicted, while only 1 sample was misclassified as belonging to the first class. The fact that only 1 out of 543 samples was incorrectly predicted demonstrates the robust performance of the YOLOv11m model.

#### 3.2.1. Seed42

The close proximity of the metrics in Table 16 across different folds indicates that the model exhibits stable and consistent performance. The use of 5-fold cross-validation and seed = 42 reveals that the model has high stability against data splitting and randomness conditions. The closeness of the training and validation results indicates that there is no overfitting and that the model generalizes well.

5-fold cross-validation results demonstrate that the YOLOv11m model exhibits high and consistent performance in both training and validation phases. Metrics close to 0.99 and low standard deviations reveal the model’s stability across different folds. Furthermore, the high performance in the holdout set indicates that the model has a strong generalization capacity not only on training data but also on unfamiliar data. Table 17 shows the performance summary of YOLOv11m model (seed = 42) under Cross-Validation and Holdout Settings.

#### 3.2.2. Seed123

The close proximity of the metrics in Table 18 across different folds indicates that the model exhibits stable and consistent performance. The use of 5-fold cross-validation and seed = 123 reveals that the model has high stability against data splitting and randomness conditions. The closeness of the training and validation results indicates that there is no overfitting and that the model generalizes well.

5-fold cross-validation results demonstrate that the YOLOv11m model exhibits high and consistent performance in both training and validation phases. Metrics close to 0.99 and low standard deviations reveal the model’s stability across different folds. Furthermore, the high performance in the holdout set indicates that the model has a strong generalization capacity not only on training data but also on unfamiliar data. Table 19 shows performance summary of YOLOv11m model (seed = 123) under Cross-Validation and Holdout Settings.

#### 3.2.3. Seed456

The close proximity of the metrics in Table 20 across different folds indicates that the model exhibits stable and consistent performance. The use of 5-fold cross-validation and seed = 456 reveals that the model has high stability against data splitting and randomness conditions. The closeness of the training and validation results indicates that there is no overfitting and that the model generalizes well.

Five-fold cross-validation results demonstrate that the YOLOv11m model exhibits high and consistent performance in both training and validation phases. Metrics close to 0.99 and low standard deviations reveal the model’s stability across different folds. Furthermore, the high performance in the holdout set indicates that the model has a strong generalization capacity not only on training data but also on unfamiliar data. Table 21 shows the performance summary of YOLOv11m model (seed = 456) under Cross-Validation and Holdout Settings.

### 3.3. Results for YOLOv12m

**Figure 15:**
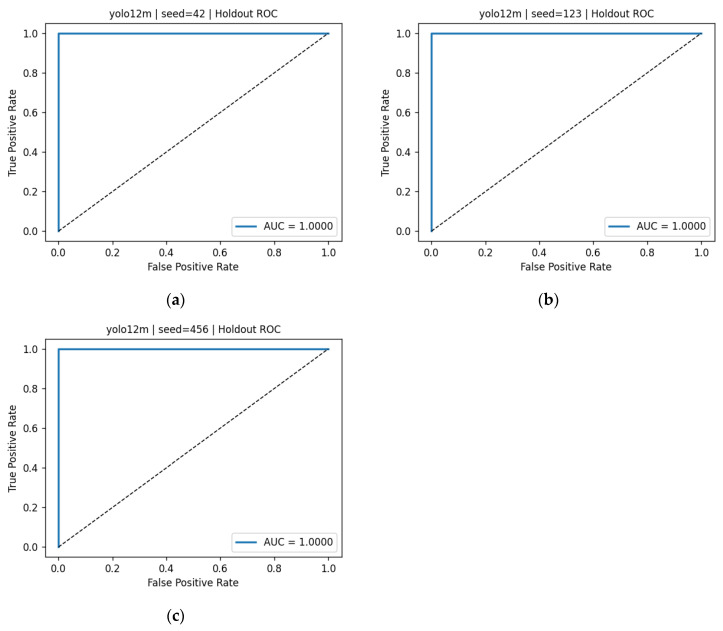
(**a**) ROC curve of the YOLOv12m on the hold-out test dataset (seed = 42); (**b**) ROC curve of the YOLOv12m on the hold-out test dataset (seed = 123); (**c**) ROC curve of the YOLOv12m on the hold-out test dataset (seed = 456).

Figure 15 shows the ROC curves obtained in the holdout test dataset for three different random seed values (42, 123, and 456) of the YOLOv12m model. Analysis of the ROC curves reveals that the YOLOv12m model yields similar results under three different random seed values. The fact that the curves have a shape similar to that of an ideal classifier indicates a high true positive rate with a low false positive rate. Additionally, the fact that the AUC values are close to their maximum values indicates that the model can distinguish between classes with high accuracy.

Table 22 presents the results obtained separately for each seed value of YOLOv12m. The fact that all evaluation criteria were obtained as 1.00 for Seed = 42 indicates that the model exhibited flawless classification performance in the relevant run. The performance criteria being at the 0.99 level for the other two seed values shows that the model’s success was maintained at a very high level. The extremely small differences between the results demonstrate that the YOLOv12m model is minimally affected by random initialization conditions and offers stable, reliable, and generalizable performance.

Table 23 shows the mean performance of the results obtained from different baseline values for the YOLOv12m model. High accuracy, sensitivity, recall, F1-score, specificity, and AUC-ROC values, along with very low standard deviation, indicate that the YOLOv12m model exhibits successful and balanced classification performance in the test dataset.

**Figure 16:**
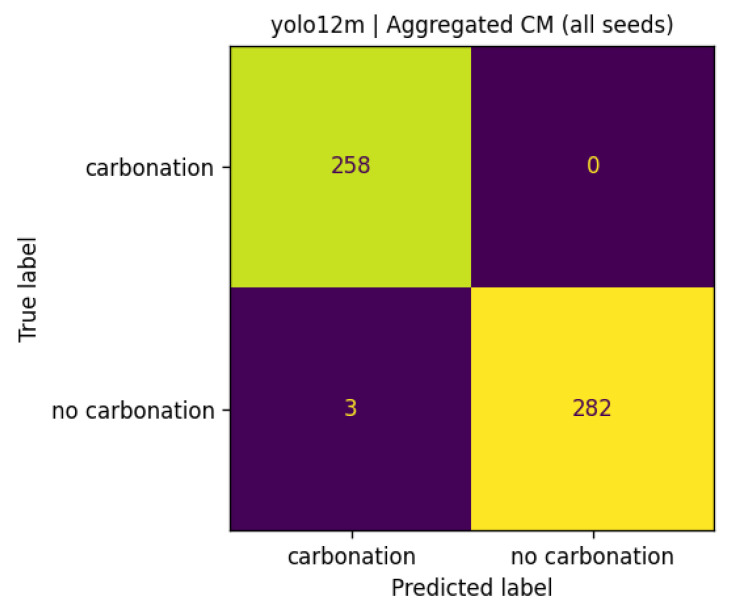
Aggregated confusion matrix obtained from repeated experiments using different random seeds (42, 123, 456) for YOLOv12m.

Figure 16 shows the aggregate confusion matrix obtained from repeated experiments using different random seed values (42, 123, 456) for the YOLOv12m model. According to the matrix values in the two-class classification problem, all 258 samples belonging to the first class were correctly classified, with no misclassification observed. In the second class, 282 samples were correctly predicted, while only 3 samples were misclassified as belonging to the first class. The results show that the YOLOv12m model achieved high success in both classes. With only 3 misclassifications out of 543 samples, the model demonstrates reliable and robust performance.

#### 3.3.1. Seed42

The close proximity of the metrics in Table 24 across different folds indicates that the model exhibits stable and consistent performance. The use of 5-fold cross-validation and seed = 42 reveals that the model has high stability against data splitting and randomness conditions. The closeness of the training and validation results indicates that there is no overfitting and that the model generalizes well.

5-fold cross-validation results demonstrate that the YOLOv12m model exhibits high and consistent performance in both training and validation phases. Metrics close to 0.98–0.99 and low standard deviations reveal the model’s stability across different folds. Furthermore, the high performance in the holdout set indicates that the model has a strong generalization capacity not only on training data but also on unfamiliar data. Table 25 shows the performance summary of YOLOv12m model (seed = 42) under Cross-Validation and Holdout Settings.

#### 3.3.2. Seed123

The close proximity of the metrics in Table 26 across different folds indicates that the model exhibits stable and consistent performance. The use of 5-fold cross-validation and seed = 123 reveals that the model has high stability against data splitting and randomness conditions. The closeness of the training and validation results indicates that there is no overfitting and that the model generalizes well.

Five-fold cross-validation results demonstrate that the YOLOv12m model exhibits high and consistent performance in both training and validation phases. Metrics close to 0.98–0.99 and low standard deviations reveal the model’s stability across different folds. Furthermore, the high performance in the holdout set indicates that the model has a strong generalization capacity not only on training data but also on unfamiliar data. Table 27 shows the performance summary of YOLOv12m model (seed = 123) under Cross-Validation and Holdout Settings.

#### 3.3.3. Seed456

The close proximity of the metrics in Table 28 across different folds indicates that the model exhibits stable and consistent performance. The use of 5-fold cross-validation and seed = 456 reveals that the model has high stability against data splitting and randomness conditions. The closeness of the training and validation results indicates that there is no overfitting and that the model generalizes well.

Five-fold cross-validation results demonstrate that the YOLOv12m model exhibits high and consistent performance in both training and validation phases. Metrics close to 0.98–0.99 and low standard deviations reveal the model’s stability across different folds. Furthermore, the high performance in the holdout set indicates that the model has a strong generalization capacity not only on training data but also on unfamiliar data. Table 29 shows the performance summary of YOLOv12m model (seed = 456) under Cross-Validation and Holdout Settings.

### 3.4. Results for YOLOv26m

**Figure 17:**
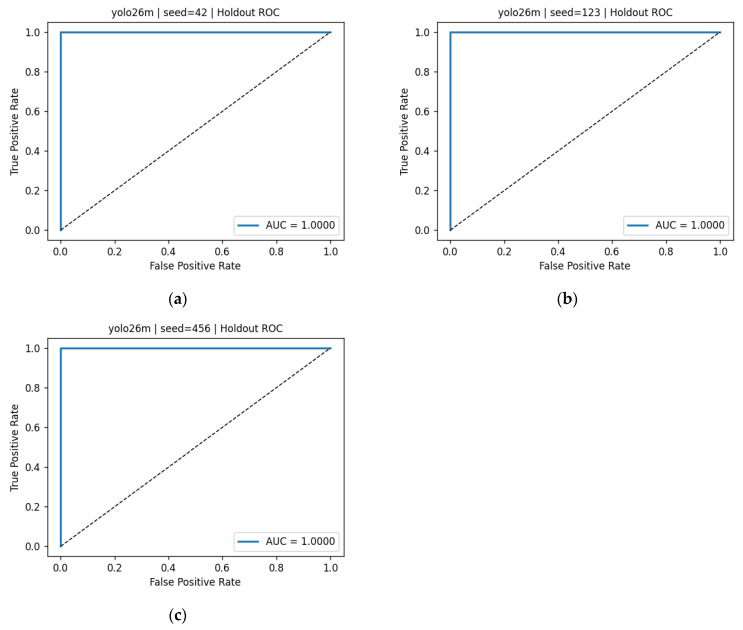
(**a**) ROC curve of the YOLOv26m on the hold-out test dataset (seed = 42); (**b**) ROC curve of the YOLOv26m on the hold-out test dataset (seed = 123); (**c**) ROC curve of the YOLOv26m on the hold-out test dataset (seed = 456).

Figure 17 shows the ROC curves obtained in the holdout test dataset for three different random seed values (42, 123, and 456) of the YOLOv26m model. The obtained ROC curves show that the YOLOv26m model offers strong classification performance regardless of the seed value. The fact that the curves were positioned near the upper left region in all three experiments indicates that the model is successful in terms of sensitivity and selectivity. The AUC values of 1.00 support the idea that the model produces reliable and consistent results.

Table 30 shows the results obtained for YOLOv26m according to different seed values. The closeness of the performance metrics indicates that the model yields consistent and stable results under different random initial seed conditions. Small differences show that the model is not significantly affected by randomness.

Table 31 shows the mean performance obtained for YOLOv26m at different seed values. High accuracy, sensitivity, recall, F1 score, specificity, and AUC-ROC values with low standard deviation indicate that the model exhibits balanced and successful classification performance on the test dataset. In particular, the AUC-ROC value being close to 1 shows that it can make strong distinctions between classes.

**Figure 18:**
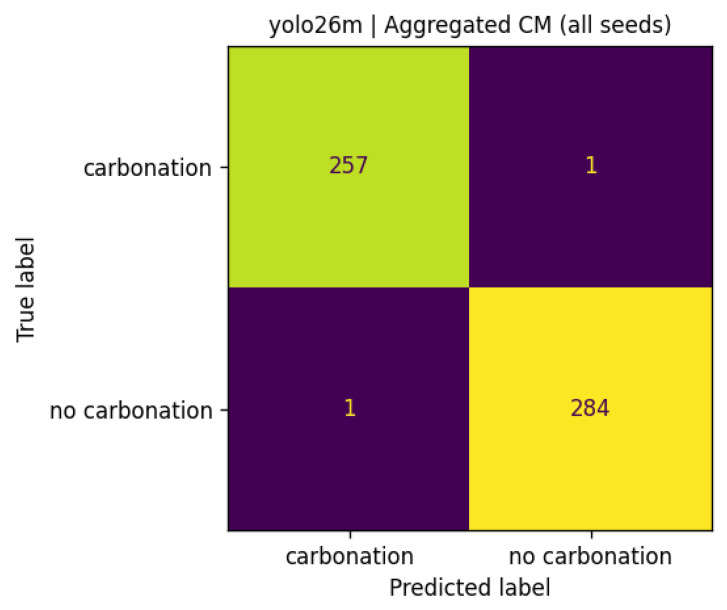
Aggregated confusion matrix obtained from repeated experiments using different random seeds (42, 123, 456) for YOLOv26m.

Figure 18 shows the overall confusion matrix obtained from repeated experiments using different random seed values (42, 123, 456) for the YOLOv26m model. According to the matrix values in the two-class classification problem, only 1 of the 258 samples belonging to the first class was misclassified. In the second class, 284 samples were correctly predicted, and only 1 sample was misclassified as belonging to the first class. The fact that only 2 out of 543 samples were incorrectly predicted shows that the model’s performance is quite robust.

#### 3.4.1. Seed42

The close proximity of the metrics in Table 32 across different folds indicates that the model exhibits stable and consistent performance. The use of 5-fold cross-validation and seed = 42 reveals that the model has high stability against data splitting and randomness conditions. The closeness of the training and validation results indicates that there is no overfitting and that the model generalizes well.

Table 33 shows the performance summary of YOLOv26m model (seed = 42) under Cross-Validation and Holdout Settings.

#### 3.4.2. Seed123

The close proximity of the metrics in Table 34 across different folds indicates that the model exhibits stable and consistent performance. The use of 5-fold cross-validation and seed = 123 reveals that the model has high stability against data splitting and randomness conditions. The closeness of the training and validation results indicates that there is no overfitting and that the model generalizes well.

Table 35 shows performance summary of YOLOv26m model (seed = 123) under Cross-Validation and Holdout Settings.

#### 3.4.3. Seed456

The close proximity of the metrics in Table 36 across different folds indicates that the model exhibits stable and consistent performance. The use of 5-fold cross-validation and seed = 456 reveals that the model has high stability against data splitting and randomness conditions. The closeness of the training and validation results indicates that there is no overfitting and that the model generalizes well.

Table 37 shows the performance summary of YOLOv26m model (seed = 456) under Cross-Validation and Holdout Settings.

### 3.5. Inference Metrics for All YOLO Models Used in the Study

Table 38 shows the inference efficiency. Using Table 38, the prediction speed, latency, and hardware usage of the models can be compared. When Table 38 is examined, it is seen that all models were tested in the same GPU environment (CUDA). The lowest average latency was obtained with the YOLOv11m model (13.88 ms), indicating that it is the fastest model in single-image estimation. Similarly, the highest frame rate (FPS = 72.02) also belongs to the YOLOv11m model. These results show that YOLOv11m is the most efficient option for real-time applications. The YOLOv8m model also performed quite well, producing results very close to YOLOv11m with an average latency of 14.30 ms and a value of 69.91 FPS. The YOLOv26m model also offered balanced performance with a latency of 14.10 ms and a value of 70.91 FPS. In contrast, the YOLOv12m model had the highest average latency (15.76 ms) and the lowest FPS (63.42), remaining slower than the other models. There are no significant differences between the models in terms of memory usage. GPU peak memory consumption ranged from approximately 152–158 MB.

In conclusion, the YOLOv11m represents the best model in terms of speed and efficiency, while the YOLOv8m and YOLOv26m represent balanced alternatives. The YOLOv12m, however, lags behind the other models in terms of speed.

**Figure 19:**
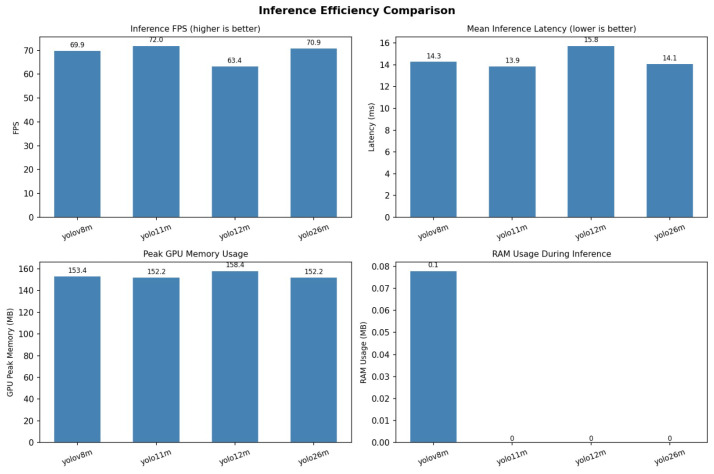
Inference efficiency comparison.

Figure 19 shows a comparison of the inference efficiency of the YOLO models. Examining Figure 19, it can be seen that YOLOv11m has the lowest average latency and is the fastest model. Similarly, it achieves the highest FPS value, providing an advantage in real-time usage. The YOLOv8m and YOLOv26m models showed performance quite close to YOLOv11m, standing out as balanced models with both low latency and high FPS values. In contrast, YOLOv12m showed higher latency and lower FPS values compared to the other models. This indicates that the model’s processing speed is relatively lower. The memory usage graph shows no significant difference between the models, with all models exhibiting similar GPU memory consumption. Overall, Figure 19 shows that YOLOv11m is the most successful model in terms of speed, while YOLOv8m and YOLOv26m are balanced alternatives.

## 4. Discussion

In this section, the performance of carbonation classification tasks carried out using different versions of the YOLO architecture (v8m, v11m, v12m, v26m) is comprehensively evaluated based on training tests conducted in the development environment. The findings reveal that increasing the complexity of the model architecture and the number of parameters leads to significant improvements in accuracy rates; however, this increase results in high costs in terms of processing time and resource utilization, especially for embedded systems.

This study comprehensively compares the performance of different YOLO-based models. When 5-fold cross-validation and independent test results are evaluated together, it is seen that the YOLOv8m and YOLOv11m models are quite close to each other in terms of all evaluation metrics (accuracy, precision, recall, F1-score, and ROC-AUC) and exhibit the highest performance. Both models achieving approximately 0.998 accuracy, 1.00 precision, and 0.998 F1-score values demonstrate the strong generalization ability of the model and the stable learning process.

In contrast, the relatively lower performance obtained in the YOLOv12m and YOLOv26m models, especially in recall and F1-score metrics, suggests that these models may have a more limited capacity to adapt to the characteristics of the dataset. This situation suggests that architectural differences or optimization behaviors may affect the balance between classes.

When evaluated in terms of model selection, the similar performance obtained with YOLOv8m and YOLOv11m confirms that the proposed approach produces consistent and reliable results even with more up-to-date architectures.

An early stopping mechanism was implemented in the training of all models to prevent overfitting. The maximum number of training sessions was set at 50 epochs, and the patience parameter was defined as 10 for situations where no improvement was observed. Thanks to this approach, the training process is automatically terminated if no improvement in validation performance is seen for 10 consecutive epochs. This ensures that the models stop at the point where they provide the best generalization performance not only to the training data but also to the validation data, minimizing the risk of overfitting.

The inference efficiency results of the YOLO models used in the study showed some differences among the evaluated YOLO models. Although all models were tested under the same hardware conditions, YOLOv11m stood out as the most efficient model for real-time applications, achieving the lowest average latency and the highest FPS. YOLOv8m and YOLOv26m models also yielded results quite close to YOLOv11m. The fact that both models offer low latency and high processing speed indicates a balanced structure between performance and computational cost. Therefore, they can be considered suitable alternatives in applications with limited resources. In contrast, YOLOv12m had the highest latency and the lowest FPS. This may be due to the model having more complex computational steps or being less optimized in terms of architecture during the inference process.

Notably, GPU peak memory usage remained at similar levels across all models. This finding suggests that the speed differences between the models are related more to computational efficiency than to memory consumption. Overall, the results show that the YOLOv11m model is the most advantageous option in terms of speed and efficiency, while the YOLOv8m and YOLOv26m models offer strong alternatives.

The dataset used in this study was obtained under controlled laboratory conditions and does not include some variables that may be encountered in the field, such as lighting variations, different camera angles, and partial carbonation. While this allowed the model performance to be evaluated on a homogeneous and controlled data distribution, it poses a limitation in terms of generalizability to real field conditions. Future studies aim to increase the robustness of the model by using more heterogeneous datasets that reflect field conditions.

The high accuracy obtained indicates strong model performance, but it should also be evaluated in terms of the structural characteristics of the dataset. The limited diversity of the dataset used may have facilitated the model’s learning process. This can lead to overly optimistic performance results, particularly in homogeneous datasets. However, in this study, the training and validation sets were carefully separated to minimize the risk of data leakage, and a cross-validation method was used in model evaluation. Nevertheless, it is stated that further studies on larger and more diverse datasets will reveal the generalizability capacity of the model more comprehensively.

In general, the YOLO-based classification models offer applicable, practical, and effective solutions in automation systems with their architectures that provide a balance between resource efficiency and accuracy for classifying carbonation in concrete images. Table 39 compares the model, aim, and results of recent studies using artificial intelligence methods on carbonation.

As shown in Table 39, while most studies focus on estimating the depth of carbonation in concrete samples, this study focuses on classifying whether carbonation is present or absent. Furthermore, while artificial neural networks and deep learning models are commonly used methods, YOLO-based classification models were preferred in this study. The results show that the 100% accuracy obtained is comparable to prediction models in the literature.

These results were obtained within the controlled and relatively homogeneous structure of the dataset. This indicates that, due to the relatively homogeneous structure and limited diversity of the dataset, model performance may not be guaranteed at the same level for different data distributions, and this is a limitation of the study in terms of generalizability.”

To verify the reliability and reproducibility of the high accuracy values reported in the study, the dataset was split into 80% training and 20% holdout, and training was conducted with 80% split set using the 5-fold cross-validation method. In addition, to evaluate the model’s sensitivity to initial weights and randomness in data splitting, all experiments were repeated under three different seed values (42, 123, and 456). The results showed no significant performance change between different seed values and that the model outputs demonstrated high consistency. Furthermore, the performance results obtained on the holdout test set were consistent with the cross-validation results, confirming that the model exhibited stable performance regardless of the data splitting strategy. These findings indicate that the reported high accuracy values are not due to data leakage; rather, the model performs generalizable and consistent learning on the dataset.

## 5. Conclusions

The study aimed to develop a robust and field-applicable deep learning-based classification framework for the automated detection of carbonation presence on concrete surfaces using images, while systematically comparing the performance of different YOLO architectures. Specifically, the objectives of the study were: (i) to evaluate the capability of multiple state-of-the-art YOLO variants in accurately distinguishing carbonated and non-carbonated concrete surfaces, (ii) to analyze the trade-offs between accuracy, computational efficiency, and model complexity for practical deployment scenarios, and (iii) to assess the suitability of a previously unused dataset for carbonation classification tasks in civil engineering applications.

Experimental results show that the YOLOv8m and YOLOv11m models emerged as the most successful, achieving 100% accuracy. The YOLOv12m and YOLOv26m models were noteworthy for their stable performance and high generalizability, particularly under varying environmental conditions; however, while not achieving the highest accuracy levels, they also stood out as stable alternatives. Inference efficiency analyses revealed differences in speed among the YOLO models. YOLOv11m stood out as the most suitable model for real-time applications with the lowest latency and highest FPS. While YOLOv8m and YOLOv26m offered strong alternatives with balanced performance, YOLOv12m lagged behind the other models in terms of speed. Furthermore, similar memory usage values indicated that the performance differences stemmed primarily from computational efficiency. The findings suggest that different YOLO versions should be evaluated based on multi-dimensional criteria such as detection accuracy and processing time.

The ConcreteCARB dataset [37] used in this study was collected under controlled laboratory conditions. Therefore, it may not fully reflect carbonation processes in structures exposed to real-world conditions. Additionally, the dataset owners [37] noted that differences in lighting, shooting distance, and angle may be observed as the images were obtained with mobile phone cameras. The dataset only includes surface-level carbonation and a binary classification (Carbonated/No Carbonation), and does not cover intermediate or partial states [37]. Given the dynamic nature of environmental conditions, it remains unclear to what extent the model can adapt to environmental variations. Despite these limitations, ConcreteCARB dataset appeared as a suitable dataset for carbonation classification tasks in civil engineering applications.

The usability of YOLOv11m architecture should be tested not only on concrete but also on other materials with similar visual complexity, and its generalizability capacity should be increased with datasets from different regions. Faster adaptation to different geographical and environmental conditions can be achieved by integrating transfer learning strategies. Energy efficiency and hardware compatibility analyses are important for the sustainability of systems that can operate in real-time in the field. Since the dataset used was obtained from a single region, creating larger and more diversified datasets reflecting different climate and light conditions will enable the model to gain a stronger generalization capacity.

Model training was performed using images obtained under controlled conditions. This may limit the model’s ability to generalize to different lighting, angles, and surface variations in real-world field conditions. Future works aim to improve model performance through advanced data enhancement methods and systematic hyperparameter optimization.

## Figures and Tables

**Table 1:** YOLO Models used in this study [50,51,52,53].

Version	Year	Tasks	Framework
YOLOv8	2023	Object Detection, Instance Segmentation, Classification, Pose/Keypoints, Oriented Detection	Pytorch
YOLOv11	2024	Object Detection, Instance Segmentation, Classification, Pose/Keypoints, Oriented Detection	Pytorch
YOLOv12	2025	Object Detection, Segmentation, Classification, Pose Estimation, and Oriented Bounding Box Detection	PyTorch
YOLOv26	2026	Object Detection, Segmentation, Classification, Pose Estimation, and Oriented Bounding Box Detection	PyTorch

YOLO models were implemented using the Ultralytics YOLO framework. Ultralytics is an open-source Python library widely used in object detection and computer vision, particularly for easily implementing YOLO algorithms. YOLO’s different-sized models vary in terms of computing power requirements and accuracy levels. Models such as Nano, Small, Medium, Large, and Xlarge offer different advantages depending on their size. Model choice depends on application needs and device resources. In this study, the “m” version (medium) of the YOLOv8, YOLOv11, YOLOv12, and YOLOv26 deep learning models from among the YOLO versions was used as the deep learning models.

**Table 2:** Features and differences between YOLO models [58].

Model	Features and Differences
YOLOv8	- It uses the C2f block in the backbone. Anchor-free decoupled head enables a unified multitasking sensing framework.
YOLOv11	- It uses the C3k2 block instead of the C2f block in the spine. - It adds the C2PSA block, which allows the model to focus on important areas. - It also supports tasks other than object detection (segmentation, pose estimation, etc.).
YOLOv12	- It offers an attention-centered approach as an alternative to CNN-based architectures and utilizes the A2 module. - It uses R-ELAN to solve the overhead and optimization problems brought about by the attention mechanism. - It requires FlashAttention technology to overcome the memory inefficiency of the attention mechanism.
YOLOv26	- CSP-Muon (Edge-Optimized CNN). - Endpoint optimized, DFL-free learning; one-to-one label assignment; detection header without native NMS for low-latency deployment; optimized for CP.

**Table 3:** Confusion matrix.

Actual/Predicted	Positive (P)	Negative (N)
Positive (P)	True Positive (TP)	False Negative (FN)
Negative (N)	False Positive (FP)	True Negative (TN)

**Table 4:** Model training configuration.

Hyperparameter	Mean	Value
*General Training Parameters*		
task	Type of task to be performed	classify
mode	Operating mode	train
model	Model file used	/root/carbonation_cls_yolo_study/yolov8m-cls.pt,/root/carbonation_cls_yolo_study/yolo11m-cls.pt, yolo12m-cls.yaml,/root/carbonation_cls_yolo_study/yolo26m-cls.pt
data	Dataset YAML file	data
epochs	Number of training cycles	50
time	Time limit	null
patience	Number of epochs waiting for early termination	10
batch	Batch size	64
imgsz	Input image size	640
device	GPU/CPU selection	null
workers	Number of data loader processors	0
name	Test name	train
pretrained	Pre-trained weight usage	true
optimizer	Optimization algorithm	auto
*Save/File Settings*		
save	Save the training output	true
save_period	How many epochs does it take to register a model?	−1
cache	Save images to RAM/Disk	false
project	Results folder	project
name	Test name	name
exist_ok	If the same folder already exists, overwrite it.	true
resume	Continue interrupted training	false
*Repeatability*		
seed	Seed of randomness	42, 123, 456
deterministic	The results must be reproducible	true
*Data Structure/Class Settings*		
single_cls	Treat all classes as one class	false
classes	Training with specific classes	null
val	Perform validation	true
split	train/val/test split	val
save_json	The model saves the object results it detects to a file in JSON (JavaScript Object Notation) format	false
*Learning Rate and Training Dynamics*		
lr0	Initial learning rate	0.01
lrf	Final learning rate	0.01
momentum	Coefficient of momentum	0.937
weight_decay	Weight punishment	0.0005
warmup_epochs	Warmup epoch number	3
warmup_momentum	Warmup momentum	0.8
warmup_bias_lr	Warmup bias learning rate	0.1
cos_lr	Cosine learning rate scheduler	false
*Loss Function Coefficients*		
box	Bounding box loss coefficient	7.5
cls	Classification loss coefficient	0.5
dfl	Distribution Focal Loss Coefficient	1.5
pose	Pose estimation loss	12
kobj	Keypoint objectness loss	1
*Augmentation*		
hsv_h	Hue change	0.015
hsv_s	Saturation change	0.7
hsv_v	Brightness change	0.4
degrees	Rotate	0
translate	Random horizontal/vertical panning ratio of the image during training	0.1
scale	Scaling	0.5
shear	The amount of cropping/sliding transformation applied to the image during training	0
perspective	Perspective shift	0
close_mosaic	Closing mosaics in later epochs	10
auto_augment	Apply an automated data enhancement strategy to the images	randaugment
*Inference Parameters*		
conf	Confidence score threshold	null
iou	NMS IoU threshold	0.7
max_det	Maximum number of detections	300
augment	Test-time augmentation	false
agnostic_nms	Class-independent NMS	false
retina_masks	High-quality masks	false
*Output Visualization*		
plots	Generate graphic	false
show	Show on screen	false
show_labels	Show labels	true
show_conf	Show confidence score	true
show_boxes	Show boxes	true
line_width	Box line thickness	null
*Export/Deployment*		
format	ONNX, TorchScript, etc.	torchscript
keras	TensorFlow/Keras export	false
optimize	Apply optimization	false
int8	INT8 quantization	false
dynamic	Dynamic input	false
simplify	Simplify ONNX	true
opset	ONNX opset version	null
workspace	TensorRT workspace	null
nms	Add NMS during export	false
*Performance/Hardware*		
amp	Mixed precision training	true
half	FP16 inference	false
dnn	OpenCV DNN backend	false
profile	PyTorch compile	false
compile	Speed profile	false
*Segmentation Parameters*		
overlap_mask	Allow the masks to overlap	true
mask_ratio	Mask resolution ratio	4
*Others*		
freeze	Layered freeze	null
multi_scale	Multi-scale training	0
dropout	Dropout rate	0.0
end2end	End-to-end inference	null
vid_stride	Video frame skipping	1
save_frames	Save video frames	false
save_txt	txt output	false
save_conf	Save confidence score	false
embed	Generate feature vector	null
stream_buffer	Stream memory	false
verbose	Provides detailed output	false
rect	Rectangular training	false
fraction	Data usage rate	1
source	Input source	null
visualize	Feature map representation	false
rle	Run-Length Encoding	1
angle	Object angle information	1
nbs	Nominal batch size	64
flipud	Rotates the image vertically	0
fliplr	Rotates the image horizontally	0.5
bgr	Red-Green-Blue	0
mosaic	Combines the images	1.0
mixup	Mixes the images	0
cutmix	Inserts a segment of an image into another image	0
erasing	Removes random areas from the image	0.4
cfg	Model configuration file	null

**Table 5:** Comparison of YOLO models used in the classification task.

Model	Accuracy (Mean)	Accuracy (Std)	Precision (Mean)	Precision (Std)	Recall (Mean)	Recall (Std)	F1 Score (Mean)	F1 Score (Std)	Specificity (Mean)	Specificity (Std)	AUC-ROC (Mean)	AUC-ROC (Std)
YOLOv8m	0.9981	0.0026	1	0	0.9964	0.0049	0.9982	0.0024	1	0	1	0
YOLOv11m	0.9981	0.0026	1	0	0.9964	0.0049	0.9982	0.0024	1	0	1	0
YOLOv12m	0.9944	0.0045	1	0	0.9894	0.0085	0.994	0.0043	1	0	1	0
YOLOv26m	0.9963	0.0026	0.9965	0.00491	0.9964	0.0049	0.9964	0.0024	0.9961	0.0054	1	0

**Table 6:** Final performance metrics (Per Seed Holdout) (YOLOv8m).

Model	Accuracy	Precision	Recall	F1 Score	Specificity	AUC-ROC
Seed = 42	1.0	1.0	1.0	1.0	1.0	1.0
Seed = 123	0.9944	1.0	0.9894	0.9947	1.0	1.0
Seed = 456	1.0	1.0	1.0	1.0	1.0	1.0

**Table 7:** Final performance metrics (Aggregated Holdout) (YOLOv8m).

Model	Accuracy (Mean)	Accuracy (Std)	Precision (Mean)	Precision (Std)	Recall (Mean)	Recall (Std)	F1 Score (Mean)	F1 Score (Std)	Specificity (Mean)	Specificity (Std)	AUC-ROC (Mean)	AUC-ROC (Std)
Aggregated Holdout	0.9981	0.0026	1.0	0.0	0.9964	0.0049	0.9982	0.0024	1.0	0.0	1.0	0.0

**Table 8:** Performance metrics values at seed = 42 for each fold of the YOLOv8m model.

	Train						Validation					
Fold	Accuracy	Precision	Recall	F1 Score	Specificity	AUC-ROC	Accuracy	Precision	Recall	F1 Score	Specificity	AUC-ROC
1	0.9948	0.9901	1.0	0.9950	0.9891	0.9996	0.9931	0.9870	1.0	0.9934	0.9855	1.0
2	0.9930	0.9868	1.0	0.9933	0.9855	0.9999	1.0	1.0	1.0	1.0	1.0	1.0
3	0.9878	1.0	0.9768	0.9882	1.0	0.9998	0.9930	1.0	0.9866	0.9932	1.0	0.9996
4	0.9775	0.9587	1.0	0.9789	0.9528	0.9998	0.9930	0.9868	1.0	0.9933	0.9855	1.0
5	0.9930	0.9869	1.0	0.9934	0.9855	0.9981	1.0	1.0	1.0	1.0	1.0	1.0

**Table 9:** Performance summary of YOLOv8m model (seed = 42) under Cross-Validation and Holdout Settings.

		Accuracy	Precision	Recall	F1 Score	Specificity	AUC-ROC
**Cross Validation**	**Train (Mean)**	0.9892	0.9845	0.9953	0.9898	0.9826	0.9994
	**Train (Std)**	0.0063	0.0137	0.0092	0.9898	0.0157	0.0006
	**Val (Mean)**	0.9958	0.9947	0.9973	0.9960	0.9942	0.9999
	**Val (Std)**	0.0033	0.0064	0.0053	0.0032	0.0070	0.0001
	**Holdout**	1.0	1.0	1.0	1.0	1.0	1.0

**Table 10:** Performance metrics values at seed = 123 for each fold of the YOLOv8m model.

	Train						Validation					
Fold	Accuracy	Precision	Recall	F1 Score	Specificity	AUC-ROC	Accuracy	Precision	Recall	F1 Score	Specificity	AUC-ROC
1	0.9948	0.9966	0.9933	0.9950	0.9963	0.9998	1.0	1.0	1.0	1.0	1.0	1.0
2	0.9913	0.9966	0.9867	0.9916	0.9963	0.9999	0.9931	0.9870	1.0	0.9934	0.9855	0.9998
3	0.9896	1.0	0.9801	0.9899	1.0	0.9994	0.9930	1.0	0.9866	0.9932	1.0	0.9974
4	0.9948	0.9933	0.9966	0.9950	0.9927	0.9997	0.9930	0.9868	1.0	0.9933	0.9855	0.9996
5	0.9930	0.9966	0.9900	0.9933	0.9963	0.9988	1.0	1.0	1.0	1.0	1.0	1.0

**Table 11:** Performance summary of YOLOv8m model (seed = 123) under Cross-Validation and Holdout Settings.

		Accuracy	Precision	Recall	F1 Score	Specificity	AUC-ROC
**Cross Validation**	**Train (Mean)**	0.9927	0.9966	0.9893	0.9930	0.9963	0.9995
	**Train (Std)**	0.0020	0.0020	0.0056	0.0019	0.0022	0.0003
	**Val (Mean)**	0.9958	0.9947	0.9973	0.9960	0.9942	0.9993
	**Val (Std)**	0.0033	0.0064	0.0053	0.0032	0.0070	0.0009
	**Holdout**	0.9944	1.0	0.9894	0.9947	1.0	1.0

**Table 12:** Performance metrics values for the YOLOv8m model at seed = 456.

	Train						Validation					
Fold	Accuracy	Precision	Recall	F1 Score	Specificity	AUC-ROC	Accuracy	Precision	Recall	F1 Score	Specificity	AUC-ROC
1	0.9930	0.9868	1.0	0.9933	0.9855	0.9999	1.0	1.0	1.0	1.0	1.0	1.0
2	0.9948	0.9933	0.9966	0.9950	0.9927	0.9999	0.9931	0.9870	1.0	0.9934	0.9855	0.9998
3	0.9965	1.0	0.9933	0.9966	1.0	0.9999	1.0	1.0	1.0	1.0	1.0	1.0
4	0.9982	1.0	0.9966	0.9983	1.0	0.9999	1.0	1.0	1.0	1.0	1.0	1.0
5	0.9965	0.9934	1.0	0.9966	0.9927	1.0	0.9930	0.9868	1.0	0.9933	0.9855	0.9893

**Table 13:** Performance summary of YOLOv8m model (seed = 456) under Cross-Validation and Holdout Settings.

		Accuracy	Precision	Recall	F1 Score	Specificity	AUC-ROC
**Cross Validation**	**Train (Mean)**	0.9958	0.9947	0.9973	0.9960	0.9942	0.9999
	**Train (Std)**	0.0017	0.0049	0.0024	0.0016	0.0054	0.00003
	**Val (Mean)**	0.9972	0.9947	1.0	0.9973	0.9942	0.9978
	**Val (Std)**	0.0033	0.0064	0.0	0.0032	0.0070	0.0042
	**Holdout**	1.0	1.0	1.0	1.0	1.0	1.0

**Table 14:** Final performance metrics (Per Seed Holdout) (YOLOv11m).

Model	Accuracy	Precision	Recall	F1 Score	Specificity	AUC-ROC
Seed = 42	0.9944	1.0	0.9894	0.9947	1.0	1.0
Seed = 123	1.0	1.0	1.0	1.0	1.0	1.0
Seed = 456	1.0	1.0	1.0	1.0	1.0	1.0

**Table 15:** Final performance metrics (Aggregated Holdout) (YOLOv11m).

Model	Accuracy (Mean)	Accuracy (Std)	Precision (Mean)	Precision (Std)	Recall (Mean)	Recall (Std)	F1 Score (Mean)	F1 Score (Std)	Specificity (Mean)	Specificity (Std)	AUC-ROC (Mean)	AUC-ROC (Std)
Aggregated Holdout	0.9981	0.0026	1.0	0.0	0.9964	0.0049	0.9982	0.0024	1.0	0.0	1.0	0.0

**Table 16:** Performance metrics values at seed = 42 for each fold of the YOLOv11m model.

	Train						Validation					
Fold	Accuracy	Precision	Recall	F1 Score	Specificity	AUC-ROC	Accuracy	Precision	Recall	F1 Score	Specificity	AUC-ROC
1	0.9913	0.9966	0.9966	0.9916	0.9916	0.9998	1.0	1.0	1.0	1.0	1.0	1.0
2	0.9896	0.9804	1.0	0.9901	0.9782	0.9999	1.0	1.0	1.0	1.0	1.0	1.0
3	0.9809	0.9678	0.9966	0.9820	0.9637	0.9979	0.9861	0.9740	1.0	0.9868	0.9710	1.0
4	0.9671	0.9435	0.9966	0.9694	0.9347	0.9988	0.9930	0.9868	1.0	0.9933	0.9855	0.9978
5	0.9948	0.9901	1.0	0.9950	0.9891	1.0	1.0	1.0	1.0	1.0	1.0	1.0

**Table 17:** Performance summary of YOLOv11m model (seed = 42) under Cross-Validation and Holdout Settings.

		Accuracy	Precision	Recall	F1 Score	Specificity	AUC-ROC
**Cross Validation**	**Train (Mean)**	0.9847	0.9757	0.9960	0.9856	0.9724	0.9993
	**Train (Std)**	0.0099	0.0187	0.0048	0.0091	0.0218	0.0008
	**Val (Mean)**	0.9958	0.9921	1.0	0.9960	0.9913	0.9995
	**Val (Std)**	0.0055	0.0104	0.0	0.0052	0.0115	0.0008
	**Holdout**	0.9944	1.0	0.989	0.9947	1.0	1.0

**Table 18:** Performance metrics values at seed = 123 for each fold of the YOLOv11m model.

	Train						Validation					
Fold	Accuracy	Precision	Recall	F1 Score	Specificity	AUC-ROC	Accuracy	Precision	Recall	F1 Score	Specificity	AUC-ROC
1	0.9896	0.9933	0.9867	0.99	0.9927	0.9966	0.9931	1.0	0.9868	0.9933	1.0	1.0
2	0.9878	0.9966	0.9800	0.9882	0.9963	0.9995	1.0	1.0	1.0	1.0	1.0	1.0
3	0.9948	0.9901	1.0	0.9950	0.9891	0.9986	0.9930	0.9868	1.0	0.9933	0.9855	1.0
4	1.0	1.0	1.0	1.0	1.0	1.0	0.9861	0.9866	0.9866	0.9866	0.9855	0.9998
5	0.9930	0.9869	1.0	0.9934	0.9855	0.9988	1.0	1.0	1.0	1.0	1.0	1.0

**Table 19:** Performance summary of YOLOv11m model (seed = 123) under Cross-Validation and Holdout Settings.

		Accuracy	Precision	Recall	F1 Score	Specificity	AUC-ROC
**Cross Validation**	**Train (Mean)**	0.9930	0.9934	0.9933	0.9933	0.9927	0.9987
	**Train (Std)**	0.0042	0.0046	0.0084	0.0041	0.0051	0.0011
	**Val (Mean)**	0.9944	0.9947	0.9947	0.9946	0.9942	0.9999
	**Val (Std)**	0.0051	0.0064	0.0064	0.0049	0.0070	7.7294
	**Holdout**	1.0	1.0	1.0	1.0	1.0	1.0

**Table 20:** Performance metrics values for the YOLOv11m model at seed = 456.

	Train						Validation					
Fold	Accuracy	Precision	Recall	F1 Score	Specificity	AUC-ROC	Accuracy	Precision	Recall	F1 Score	Specificity	AUC-ROC
1	0.9965	0.9933	1.0	0.9966	0.9927	0.9999	1.0	1.0	1.0	1.0	1.0	1.0
2	0.9948	0.9966	0.9933	0.9950	0.9963	0.9999	0.9931	0.9870	1.0	0.9934	0.9855	0.9975
3	0.9896	0.9805	1.0	0.9901	0.9782	0.9974	1.0	1.0	1.0	1.0	1.0	1.0
4	0.9948	0.9901	1.0	0.9950	0.9891	0.9999	1.0	1.0	1.0	1.0	1.0	1.0
5	0.9930	0.9966	0.9900	0.9933	0.9963	0.9994	0.9930	1.0	0.9866	0.9932	1.0	0.9982

**Table 21:** Performance summary of YOLOv11m model (seed = 456) under Cross-Validation and Holdout Settings.

		Accuracy	Precision	Recall	F1 Score	Specificity	AUC-ROC
**Cross Validation**	**Train (Mean)**	0.9937	0.9914	0.9966	0.9940	0.9905	0.9993
	**Train (Std)**	0.0023	0.0059	0.0041	0.0022	0.0067	0.0009
	**Val (Mean)**	0.9972	0.9974	0.9973	0.9973	0.9971	0.9991
	**Val (Std)**	0.0033	0.0051	0.0053	0.0032	0.0057	0.0010
	**Holdout**	1.0	1.0	1.0	1.0	1.0	1.0

**Table 22:** Final performance metrics (Per Seed Holdout) (YOLOv12m).

Model	Accuracy	Precision	Recall	F1 Score	Specificity	AUC-ROC
Seed = 42	1.0	1.0	1.0	1.0	1.0	1.0
Seed = 123	0.9889	1.0	0.9789	0.9893	1.0	1.0
Seed = 456	0.9944	1.0	0.9894	0.9947	1.0	1.0

**Table 23:** Final performance metrics (Aggregated Holdout) (YOLOv12m).

Model	Accuracy (Mean)	Accuracy (Std)	Precision (Mean)	Precision (Std)	Recall (Mean)	Recall (Std)	F1 Score (Mean)	F1 Score (Std)	Specificity (Mean)	Specificity (Std)	AUC-ROC (Mean)	AUC-ROC (Std)
Aggregated Holdout	0.9944	0.0045	1.0	0.0	0.9894	0.0085	0.9946	0.0043	1.0	0.0	1.0	0.0

**Table 24:** Performance metrics values at seed = 42 for each fold of the YOLOv12m model.

	Train						Validation					
Fold	Accuracy	Precision	Recall	F1 Score	Specificity	AUC-ROC	Accuracy	Precision	Recall	F1 Score	Specificity	AUC-ROC
1	0.9878	0.99	0.9867	0.9883	0.9891	0.9989	1.0	1.0	1.0	1.0	1.0	1.0
2	0.9930	0.9900	0.9966	0.9933	0.9891	0.9996	0.9931	0.9870	1.0	0.9934	0.9855	0.9992
3	0.9896	0.9933	0.9867	0.9900	0.9927	0.9950	1.0	1.0	1.0	1.0	1.0	1.0
4	0.9844	0.9966	0.9735	0.9849	0.9963	0.9979	1.0	1.0	1.0	1.0	1.0	1.0
5	0.9896	0.9900	0.9900	0.9900	0.9891	0.9997	0.9722	0.9863	0.96	0.9729	0.9855	0.9963

**Table 25:** Performance summary of YOLOv12m model (seed = 42) under Cross-Validation and Holdout Settings.

		Accuracy	Precision	Recall	F1 Score	Specificity	AUC-ROC
**Cross Validation**	**Train (Mean)**	0.9889	0.9920	0.9867	0.9893	0.9913	0.9982
	**Train (Std)**	0.0028	0.0026	0.0075	0.0027	0.0028	0.0017
	**Val (Mean)**	0.9930	0.9946	0.992	0.9932	0.9942	0.9991
	**Val (Std)**	0.0107	0.0065	0.0160	0.0104	0.0070	0.0014
	**Holdout**	1.0	1.0	1.0	1.0	1.0	1.0

**Table 26:** Performance metrics values at seed = 123 for each fold of the YOLOv12m model.

	Train						Validation					
Fold	Accuracy	Precision	Recall	F1 Score	Specificity	AUC-ROC	Accuracy	Precision	Recall	F1 Score	Specificity	AUC-ROC
1	0.9844	1.0	0.9700	0.9848	1.0	0.9943	0.9862	1.0	0.9736	0.9866	1.0	0.9977
2	0.9913	1.0	0.9833	0.9916	1.0	0.9995	1.0	1.0	1.0	1.0	1.0	1.0
3	0.9809	0.9708	0.9933	0.9819	0.9673	0.9985	0.9861	0.9866	0.9866	0.9866	0.9855	0.9938
4	0.9809	0.9739	0.9900	0.9819	0.9710	0.9970	0.9930	1.0	0.9866	0.9932	1.0	0.9926
5	0.9775	0.9965	0.9602	0.9780	0.9963	0.9837	0.9930	1.0	0.9866	0.9932	1.0	0.9884

**Table 27:** Performance summary of YOLOv12m model (seed = 123) under Cross-Validation and Holdout Settings.

		Accuracy	Precision	Recall	F1 Score	Specificity	AUC-ROC
**Cross Validation**	**Train (Mean)**	0.9830	0.9882	0.9794	0.9836	0.9869	0.9946
	**Train (Std)**	0.0046	0.0130	0.0124	0.0045	0.0146	0.0057
	**Val (Mean)**	0.9916	0.9973	0.9867	0.9919	0.9971	0.9945
	**Val (Std)**	0.0051	0.0053	0.0083	0.0049	0.0057	0.0040
	**Holdout**	0.9889	1.0	0.9789	0.9893	1.0	1.0

**Table 28:** Performance metrics values for the YOLOv12m model at seed = 456.

	Train						Validation					
Fold	Accuracy	Precision	Recall	F1 Score	Specificity	AUC-ROC	Accuracy	Precision	Recall	F1 Score	Specificity	AUC-ROC
1	0.9844	0.9965	0.9734	0.9848	0.9963	0.9992	0.9862	0.9868	0.9868	0.9868	0.9855	0.9988
2	0.9913	0.9966	0.9867	0.9916	0.9963	0.9982	1.0	1.0	1.0	1.0	1.0	1.0
3	0.9826	0.9834	0.9834	0.9834	0.9818	0.9972	0.9861	1.0	0.9733	0.9864	1.0	0.9965
4	0.9878	0.9933	0.9834	0.9883	0.9927	0.9944	0.9930	1.0	0.9866	0.9932	1.0	0.9897
5	0.9809	1.0	0.9635	0.9814	1.0	0.9949	0.9930	1.0	0.9866	0.9932	1.0	0.9998

**Table 29:** Performance summary of YOLOv12m model (seed = 456) under Cross-Validation and Holdout Settings.

		Accuracy	Precision	Recall	F1 Score	Specificity	AUC-ROC
Cross Validation	Train (Mean)	0.9854	0.9939	0.9781	0.9859	0.9934	0.9968
	Train (Std)	0.0037	0.0056	0.0085	0.0036	0.0062	0.0018
	Val (Mean)	0.9916	0.9973	0.9867	0.9919	0.9971	0.9969
	Val (Std)	0.0051	0.0052	0.0084	0.0049	0.0057	0.0038
	Holdout	0.9944	1.0	0.9894	0.9947	1.0	1.0

**Table 30:** Final performance metrics (Per Seed Holdout) (YOLOv26m).

Model	Accuracy	Precision	Recall	F1 Score	Specificity	AUC-ROC
Seed = 42	0.9944	1.0	0.9894	0.9947	1.0	1.0
Seed = 123	1.0	1.0	1.0	1.0	1.0	1.0
Seed = 456	0.9944	0.9895	1.0	0.9947	0.9883	1.0

**Table 31:** Final performance metrics (Aggregated Holdout) (YOLOv26m).

Model	Accuracy (Mean)	Accuracy (Std)	Precision (Mean)	Precision (Std)	Recall (Mean)	Recall (Std)	F1 Score (Mean)	F1 Score (Std)	Specificity (Mean)	Specificity (Std)	AUC-ROC (Mean)	AUC-ROC (Std)
Aggregated Holdout	0.9963	0.0026	0.9965	0.0049	0.9964	0.0049	0.9964	0.0024	0.9961	0.0054	1.0	0.0

**Table 32:** Performance metrics values at seed = 42 for each fold of the YOLOv26m model.

	Train						Validation					
Fold	Accuracy	Precision	Recall	F1 Score	Specificity	AUC-ROC	Accuracy	Precision	Recall	F1 Score	Specificity	AUC-ROC
1	0.9948	1.0	0.9900	0.9949	1.0	0.9999	1.0	1.0	1.0	1.0	1.0	1.0
2	1.0	1.0	1.0	1.0	1.0	1.0	1.0	1.0	1.0	1.0	1.0	1.0
3	0.9878	0.9966	0.9801	0.9883	0.9963	0.9993	0.9930	0.9868	1.0	0.9933	0.9855	0.9994
4	0.9948	0.9933	0.9966	0.9950	0.9927	0.9998	0.9930	0.9868	1.0	0.9933	0.9855	0.9998
5	0.9930	0.9901	0.9966	0.9933	0.9891	0.9998	1.0	1.0	1.0	1.0	1.0	1.0

**Table 33:** Performance summary of YOLOv26m model (seed = 42) under Cross-Validation and Holdout Settings.

		Accuracy	Precision	Recall	F1 Score	Specificity	AUC-ROC
**Cross Validation**	**Train (Mean)**	0.9941	0.9960	0.9927	0.9943	0.9956	0.9998
	**Train (Std)**	0.0038	0.0038	0.0070	0.0037	0.0042	0.0002
	**Val (Mean)**	0.9972	0.9947	1.0	0.9973	0.9942	0.9998
	**Val (Std)**	0.0034	0.0064	0.0	0.0032	0.0070	0.0002
	**Holdout**	0.9944	1.0	0.9894	0.9947	1.0	1.0

**Table 34:** Performance metrics values at seed = 123 for each fold of the YOLOv26m model.

	Train						Validation					
Fold	Accuracy	Precision	Recall	F1 Score	Specificity	AUC-ROC	Accuracy	Precision	Recall	F1 Score	Specificity	AUC-ROC
1	0.9930	0.9900	0.9966	0.9933	0.9891	0.9999	0.9931	1.0	0.9868	0.9933	1.0	0.9982
2	1.0	1.0	1.0	1.0	1.0	1.0	1.0	1.0	1.0	1.0	1.0	1.0
3	0.9930	0.9901	0.9966	0.9933	0.9891	0.9999	0.9930	0.9868	1.0	0.9933	0.9855	0.9998
4	0.9861	0.9867	0.9867	0.9867	0.9855	0.9963	0.9930	1.0	0.9866	0.9932	1.0	0.9868
5	0.9930	0.9966	0.9900	0.9933	0.9963	0.9993	1.0	1.0	1.0	1.0	1.0	1.0

**Table 35:** Performance summary of YOLOv26m model (seed = 123) under Cross-Validation and Holdout Settings.

		Accuracy	Precision	Recall	F1 Score	Specificity	AUC-ROC
**Cross Validation**	**Train (Mean)**	0.9930	0.9927	0.9940	0.9933	0.9920	0.9991
	**Train (Std)**	0.0043	0.0048	0.0048	0.0041	0.0053	0.0014
	**Val (Mean)**	0.9958	0.9973	0.9947	0.9960	0.9971	0.9969
	**Val (Std)**	0.0033	0.0052	0.0064	0.0032	0.0057	0.0051
	**Holdout**	1.0	1.0	1.0	1.0	1.0	1.0

**Table 36:** Performance metrics values for the YOLOv26m model at seed = 456.

	Train						Validation					
Fold	Accuracy	Precision	Recall	F1 Score	Specificity	AUC-ROC	Accuracy	Precision	Recall	F1 Score	Specificity	AUC-ROC
1	0.9878	0.9867	0.9900	0.9883	0.9855	0.9988	0.9931	0.9870	1.0	0.9934	0.9855	0.9996
2	0.9878	0.9803	0.9966	0.9884	0.9782	0.9996	0.9793	0.9620	1.0	0.9806	0.9565	0.9996
3	0.9930	0.9869	1.0	0.9934	0.9855	0.9999	1.0	1.0	1.0	1.0	1.0	1.0
4	0.9965	0.9934	1.0	0.9966	0.9927	1.0	1.0	1.0	1.0	1.0	1.0	1.0
5	0.9982	0.9966	1.0	0.9983	0.9963	1.0	0.9930	0.9868	1.0	0.9933	0.9855	0.9996

**Table 37:** Performance summary of YOLOv26m model (seed = 456) under Cross-Validation and Holdout Settings.

		Accuracy	Precision	Recall	F1 Score	Specificity	AUC-ROC
**Cross Validation**	**Train (Mean)**	0.9927	0.9888	0.9973	0.9930	0.9876	0.9996
	**Train (Std)**	0.0043	0.0056	0.0038	0.0041	0.0063	0.0004
	**Val (Mean)**	0.9930	0.9871	1.0	0.9934	0.9855	0.9997
	**Val (Std)**	0.0075	0.987	0.0	0.0070	0.0158	0.0001
	**Holdout**	0.9944	0.9895	1.0	0.9947	0.9883	1.0

**Table 38:** Inference metrics.

Model	Mean Latency (ms)	Std Latency (ms)	Min Latency (ms)	Max Latency (ms)	FPS ^1^	RAM_Usage (MB)	GPU Peak (MB)	Device
YOLOv8m	14.3026	1.6152	13.3695	20.5353	69.9169	0.07812	153.3965	CUDA
YOLOv11m	13.8840	1.0431	13.4266	22.3221	72.0253	0	152.2153	CUDA
YOLOv12m	15.7663	0.8128	15.3774	22.0508	63.4263	0	158.3813	CUDA
YOLOv26m	14.1020	1.4171	13.3621	21.8407	70.9117	0	152.2153	CUDA

^1^ Frames Per Second.

**Table 39:** Comparison with similar studies in the literature using artificial intelligence.

Study	Year	Aim	Model	Results
Lee et al. [29]	2020	Predict the carbonation behavior of concrete	Deep Neural Network	carbonation rate coefficient = 0.01 mm/(year)^1/2^
Giulietti et al. [30]	2021	Determine the carbonation depth	Convolutional Neural Networks	R^2^ = 0.96
Chen et al. [31]	2022	Predict the concrete carbonation depth	combining artificial neural network (ANN) and support vector machine (SVM)	r = 0.9788–0.9946
Tran et al. [32]	2023	Predict the carbonation depth of concrete	XGB, GB, RF, SVM	R^2^ = 0.9770, RMSE = 2.2725, MAE = 1.5218
Ehsani et al. [33]	2024	Predict the carbonation depth	MOEA/D-ANN	R^2^ = 0.947
Ma et al. [34]	2025	Predict the carbonation percentage	KNN, BP-ANN, DT, RF, GB, XGB	R^2^ = 0.974, RMSE = 1.521
Liu et al. [35]	2026	Predict the carbonation depth	RF, ANN, CNN and two enhanced models with feature interactions, carbonation equations, and attention mechanisms (ATT-ANN, ATT-CNN).	R^2^ = 0.9142
Jafari and Dorafshan [36]	2026	Determine the concrete surface carbonation	K-means clustering	90% IoU accuracy
This study	2026	Classification of concrete carbonation	YOLOv8m, YOLOv11m, YOLOv12m, YOLOv26m	Accuracy = 0.9981, Precision = 1, Recall = 0.9964, Specificity = 1, AUC-ROC = 1

## Data Availability

The original contributions presented in this study are included in the article. Further inquiries can be directed to the corresponding authors.

## Supplementary Materials

The following supporting information can be downloaded at: https://www.mdpi.com/article/10.3390/ma19112198/s1.
