# Supplementary material for: Image-Based Classification of Concrete Carbonation Using YOLO Models
Source: Materials (Basel). 2026 May 23;19(11):2198. doi: 10.3390/ma19112198 (PMC13258704; doi:10.3390/ma19112198)
Supplement: Supplementary file 1 [file materials-19-02198-s001.zip › materials-4275006-supplementary.pdf]

Supplementary Material

Table S1. Performance metrics values for the YOLOv8m model at seed=42.

	epoch	time	train/loss	metrics/accuracy_top1	val/loss	lr/pg0	lr/pg1	lr/pg2
YOLOv8m (seed42_fold1)	1	50.6883	0.67535	0.8069	0.60132	0.00015	0.00015	0.00015
	5	251.357	0.06897	0.9931	0.0443	0.000752	0.000752	0.000752
	10	503.402	0.04264	0.9931	0.02236	0.001356	0.001356	0.001356
	14	705.095	0.50448	0.74483	4.10156	0.001238	0.001238	0.001238
YOLOv8m (seed42_fold2)	1	52.8774	0.67329	0.76552	0.6189	0.00015	0.00015	0.00015
	5	264.82	0.07475	0.91724	0.27954	0.000752	0.000752	0.000752
	10	527.336	0.12227	0.97931	0.19843	0.001356	0.001356	0.001356
	13	684.885	0.03049	0.97931	0.04811	0.001271	0.001271	0.001271
YOLOv8m (seed42_fold3)	1	53.3528	0.66899	0.84028	0.58301	0.00015	0.00015	0.00015
	5	265.556	0.04396	0.97917	0.0475	0.000752	0.000752	0.000752
	10	532.949	0.43539	0.72222	0.70264	0.001356	0.001356	0.001356
	14	745.56	0.41274	0.99306	0.02018	0.001238	0.001238	0.001238
YOLOv8m (seed42_fold4)	1	53.2138	0.66767	0.82639	0.58545	0.00015	0.00015	0.00015
	5	263.94	0.06195	0.99306	0.00918	0.000752	0.000752	0.000752
	10	528.811	0.32802	0.59722	1.25684	0.001356	0.001356	0.001356
	13	686.562	0.07073	0.98611	0.01948	0.001271	0.001271	0.001271
YOLOv8m (seed42_fold5)	1	53.1686	0.66564	0.90278	0.57642	0.00015	0.00015	0.00015
	5	263.756	0.04187	0.98611	0.0168	0.000752	0.000752	0.000752
	10	528.606	0.38197	0.97917	0.05173	0.001356	0.001356	0.001356
	14	741.541	0.7256	0.97222	0.15749	0.001238	0.001238	0.001238

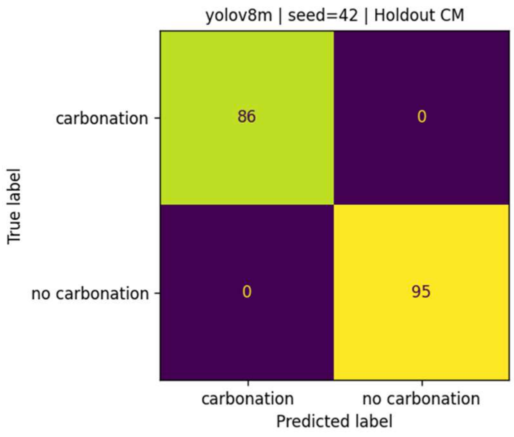

Figure S1. Confusion matrix of the YOLOv8m on the independent hold-out test set (seed=42).

Table S2. Performance metrics values for the YOLOv8m model at seed=123.

	epoch	time	train/loss	metrics/accuracy_top1	val/loss	lr/pg0	lr/pg1	lr/pg2
YOLOv8m (seed123_fold1)	1	53.3956	0.66488	0.73103	0.60645	0.00015	0.00015	0.00015
	5	264.549	0.05953	0.95172	0.0845	0.000752	0.000752	0.000752
	10	527.389	0.17671	0.61379	0.69165	0.001356	0.001356	0.001356
	14	739.406	0.33993	0.95172	0.24927	0.001238	0.001238	0.001238
YOLOv8m (seed123_fold2)	1	54.4033	0.67273	0.71034	0.6123	0.00015	0.00015	0.00015
	5	269.773	0.06462	0.9931	0.05261	0.000752	0.000752	0.000752
	10	538.104	0.13965	0.96552	0.11546	0.001356	0.001356	0.001356
	14	754.071	0.30326	0.97241	0.07943	0.001238	0.001238	0.001238
YOLOv8m (seed123_fold3)	1	55.1741	0.66491	0.72917	0.6106	0.00015	0.00015	0.00015
	5	270.719	0.07397	0.97917	0.06691	0.000752	0.000752	0.000752
	10	538.764	0.17125	0.99306	0.02327	0.001356	0.001356	0.001356
	15	809.325	0.4075	0.68056	0.43384	0.001205	0.001205	0.001205
	18	971.014	0.12276	0.86111	0.24371	0.001106	0.001106	0.001106
YOLOv8m (seed123_fold4)	1	54.1636	0.66341	0.68056	0.62915	0.00015	0.00015	0.00015
	5	265.134	0.0366	0.97917	0.05898	0.000752	0.000752	0.000752
	10	529.005	0.09481	0.82639	0.61108	0.001356	0.001356	0.001356
	13	688.845	0.18764	0.97917	0.03475	0.001271	0.001271	0.001271
YOLOv8m (seed123_fold5)	1	53.8358	0.66149	0.72222	0.63599	0.00015	0.00015	0.00015
	5	267.56	0.0359	1	0.00184	0.000752	0.000752	0.000752
	10	533.173	0.1725	0.75	7.05859	0.001356	0.001356	0.001356
	13	693.47	0.19914	1	0.00581	0.001271	0.001271	0.001271

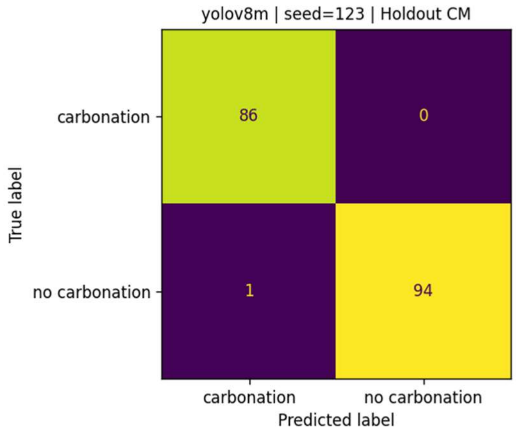

Figure S2. Confusion matrix of the YOLOv8m on the independent hold-out test set (seed=123).

Table S3. Performance metrics values for the YOLOv8m model at seed=456.

	epoch	time	train/loss	metrics/accuracy_top1	val/loss	lr/pg0	lr/pg1	lr/pg2
YOLOv8m (seed456_fold1)	1	54.0663	0.66871	0.81379	0.60669	0.00015	0.00015	0.00015
	5	266.979	0.07141	0.97931	0.10056	0.000752	0.000752	0.000752
	10	532.769	0.15779	0.95862	0.08025	0.001356	0.001356	0.001356
	15	798.063	0.09113	0.9931	0.05272	0.001205	0.001205	0.001205
	16	851.571	0.32214	0.98621	0.05128	0.001172	0.001172	0.001172
YOLOv8m (seed456_fold2)	1	54.6289	0.67652	0.77241	0.60303	0.00015	0.00015	0.00015
	5	267.743	0.04872	0.97241	0.15253	0.000752	0.000752	0.000752
	10	534.143	0.28656	0.9931	0.06499	0.001356	0.001356	0.001356
	15	798.151	0.15383	0.98621	0.1244	0.001205	0.001205	0.001205
	20	1063.75	0.19536	0.82069	0.36987	0.00104	0.00104	0.00104
YOLOv8m (seed456_fold3)	1	53.5233	0.66428	0.86806	0.57837	0.00015	0.00015	0.00015
	5	266.985	0.04165	1	0.02398	0.000752	0.000752	0.000752
	10	533.024	0.1681	0.9375	0.19919	0.001356	0.001356	0.001356
	13	693.264	0.12228	0.85417	0.35754	0.001271	0.001271	0.001271
YOLOv8m (seed456_fold4)	1	55.0238	0.66511	0.85417	0.56763	0.00015	0.00015	0.00015
	5	268.976	0.03235	1	0.00194	0.000752	0.000752	0.000752
	10	537.948	0.12762	0.99306	0.02834	0.001356	0.001356	0.001356
	13	697.823	0.13233	0.99306	0.11258	0.001271	0.001271	0.001271
YOLOv8m (seed456_fold5)	1	53.5094	0.66469	0.86806	0.55566	0.00015	0.00015	0.00015
	5	265.877	0.06624	0.97917	0.08981	0.000752	0.000752	0.000752
	10	532.961	0.19129	0.77778	1.74268	0.001356	0.001356	0.001356
	15	799.66	0.29288	0.94444	0.10817	0.001205	0.001205	0.001205
	19	1011	0.24815	0.82639	0.43225	0.001073	0.001073	0.001073

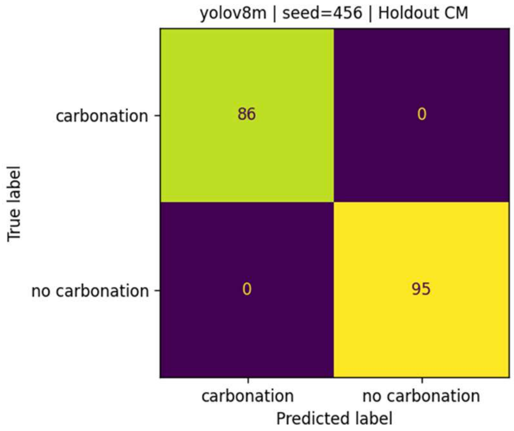

Figure S3. Confusion matrix of the YOLOv8m on the independent hold-out test set (seed=456).

Table S4. Performance metrics values for the YOLOv11m model at seed=42.

	epoch	time	train/loss	metrics/accuracy_top1	val/loss	lr/pg0	lr/pg1	lr/pg2
YOLOv11m (seed42_fold 1)	1	55.4928	0.58706	0.87586	0.34534	0.00015	0.00015	0.00015
	5	269.334	0.38635	0.77241	0.73291	0.000752	0.000752	0.000752
	10	535.815	0.30977	0.68966	6.73242	0.001356	0.001356	0.001356
	13	691.52	0.08238	1	0.00946	0.001271	0.001271	0.001271
YOLOv11m (seed42_fold 2)	1	54.4266	0.57142	0.91724	0.31335	0.00015	0.00015	0.00015
	5	268.285	0.55685	0.97241	0.03904	0.000752	0.000752	0.000752
	10	534.885	0.1306	0.92414	0.29028	0.001356	0.001356	0.001356
	12	641.643	0.61445	0.96552	0.15376	0.001304	0.001304	0.001304
YOLOv11m (seed42_fold 3)	1	54.8331	0.5862	0.92361	0.2533	0.00015	0.00015	0.00015
	5	269.399	0.095	0.97917	0.10863	0.000752	0.000752	0.000752
	10	538.073	0.2446	0.69444	4.12305	0.001356	0.001356	0.001356
	12	644.677	0.27465	0.56944	138.641	0.001304	0.001304	0.001304
YOLOv11m (seed42_fold 4)	1	53.7738	0.5645	0.97222	0.25604	0.00015	0.00015	0.00015
	5	266.581	0.06772	0.99306	0.3107	0.000752	0.000752	0.000752
	10	535.011	0.3312	0.5625	15.4062	0.001356	0.001356	0.001356
	12	642.216	0.28428	0.95139	3.5484	0.001304	0.001304	0.001304
YOLOv11m (seed42_fold 5)	1	53.8757	0.57425	0.875	0.2688	0.00015	0.00015	0.00015
	5	267.468	0.09469	0.81944	1.22192	0.000752	0.000752	0.000752
	10	533.56	0.73595	0.67361	50.7812	0.001356	0.001356	0.001356
	12	640.396	0.14837	0.89583	0.21982	0.001304	0.001304	0.001304

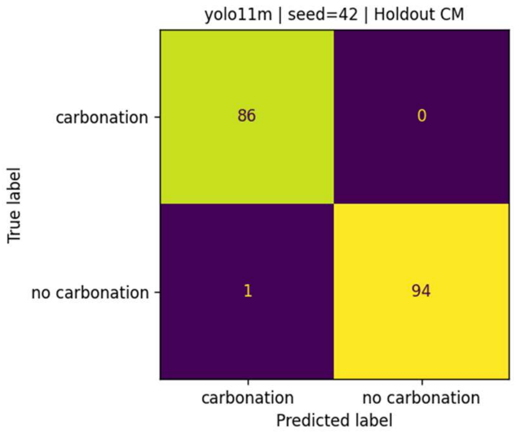

Figure S4. Confusion matrix of the YOLOv11m on the independent hold-out test set (seed=42).

Table S5. Performance metrics values for the YOLOv11m model at seed=123.

	epoch	time	train/loss	metrics/accuracy_top1	val/loss	lr/pg0	lr/pg1	lr/pg2
YOLOv11m (seed123_fold1)	1	53.8945	0.61885	0.93103	0.29761	0.00015	0.00015	0.00015
	5	269.17	0.14501	0.93103	0.12307	0.000752	0.000752	0.000752
	10	538.745	0.21337	0.67586	9.96875	0.001356	0.001356	0.001356
	14	752.09	0.6142	0.98621	0.01533	0.001238	0.001238	0.001238
YOLOv11m (seed123_fold2)	1	54.6816	0.62544	0.85517	0.35852	0.00015	0.00015	0.00015
	5	271.828	0.28637	0.71724	0.61816	0.000752	0.000752	0.000752
	10	545.153	0.19967	0.86897	1.42725	0.001356	0.001356	0.001356
	15	815.55	0.20895	0.52414	3.81445	0.001205	0.001205	0.001205
	20	1086.09	0.16222	0.97931	0.20807	0.00104	0.00104	0.00104
	24	1302.4	0.4124	0.97931	0.04742	0.000908	0.000908	0.000908
YOLOv11m (seed123_fold3)	1	53.7695	0.6137	0.96528	0.27502	0.00015	0.00015	0.00015
	5	267.583	0.0617	0.88194	0.47327	0.000752	0.000752	0.000752
	10	533.063	0.59797	0.79861	0.53223	0.001356	0.001356	0.001356
	13	693.085	0.1049	0.84722	1.8208	0.001271	0.001271	0.001271
YOLOv11m (seed123_fold4)	1	54.2773	0.62984	0.90972	0.35596	0.00015	0.00015	0.00015
	5	269.305	0.03995	0.97917	0.03931	0.000752	0.000752	0.000752
	10	537.416	0.35302	0.5	2.94238	0.001356	0.001356	0.001356
	12	644.078	0.35013	0.9375	0.28656	0.001304	0.001304	0.001304
YOLOv11m (seed123_fold5)	1	53.9487	0.61823	0.95139	0.26245	0.00015	0.00015	0.00015
	5	269.766	0.06652	0.98611	0.06332	0.000752	0.000752	0.000752
	10	538.927	0.41164	0.84722	0.35968	0.001356	0.001356	0.001356
	13	700.866	0.1953	0.9375	0.33167	0.001271	0.001271	0.001271

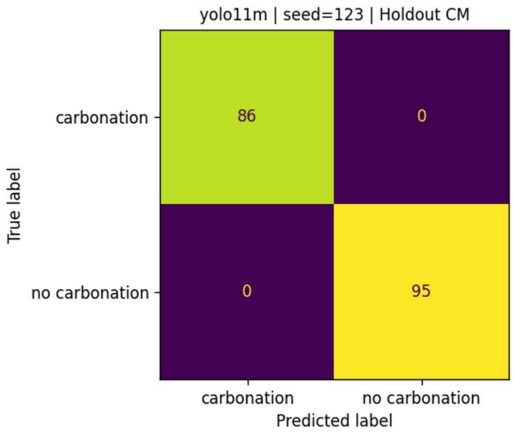

Figure S5. Confusion matrix of the YOLOv11m on the independent hold-out test set (seed=123).

Table S6. Performance metrics values for the YOLOv11m model at seed=456.

	epoch	time	train/loss	metrics/accuracy_top1	val/loss	lr/pg0	lr/pg1	lr/pg2
YOLOv11m (seed456_fold1)	1	53.5865	0.63695	0.91724	0.37073	0.00015	0.00015	0.00015
	5	267.519	0.51267	0.98621	0.02575	0.000752	0.000752	0.000752
	10	535.08	0.17331	0.81379	5.93164	0.001356	0.001356	0.001356
	13	695.923	0.17336	0.96552	0.16266	0.001271	0.001271	0.001271
YOLOv11m (seed456_fold2)	1	53.4265	0.6251	0.88966	0.46387	0.00015	0.00015	0.00015
	5	268.357	0.77597	0.97241	0.03902	0.000752	0.000752	0.000752
	10	535.258	0.19117	0.48276	4.74902	0.001356	0.001356	0.001356
	14	748.278	0.53408	0.90345	0.42557	0.001238	0.001238	0.001238
YOLOv11m (seed456_fold3)	1	54.1209	0.63924	0.95833	0.25385	0.00015	0.00015	0.00015
	5	270.038	0.08866	0.63889	2.43115	0.000752	0.000752	0.000752
	10	536.938	0.63976	0.92361	0.13663	0.001356	0.001356	0.001356
	15	803.659	0.55339	0.875	0.56482	0.001205	0.001205	0.001205
	17	910.96	0.3846	0.95139	0.21564	0.001139	0.001139	0.001139
YOLOv11m (seed456_fold4)	1	54.363	0.62873	0.95833	0.25916	0.00015	0.00015	0.00015
	5	270.226	0.0572	0.98611	0.03549	0.000752	0.000752	0.000752
	10	541.314	0.36671	0.97917	0.10658	0.001356	0.001356	0.001356
	14	756.274	0.21298	0.97917	0.17601	0.001238	0.001238	0.001238
YOLOv11m (seed456_fold5)	1	53.9332	0.6379	0.9375	0.33191	0.00015	0.00015	0.00015
	5	269.511	0.05918	0.98611	0.03937	0.000752	0.000752	0.000752
	10	540.854	0.44712	0.99306	0.04031	0.001356	0.001356	0.001356
	13	703.646	0.26016	0.97917	0.06121	0.001271	0.001271	0.001271

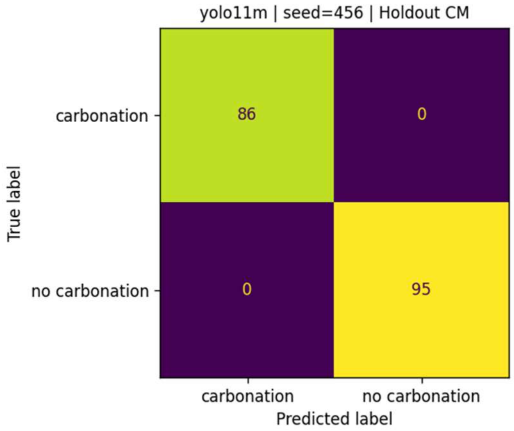

Figure S6. Confusion matrix of the YOLOv11m on the independent hold-out test set (seed=456).

Table S7. Performance metrics values for the YOLOv12m model at seed=42.

	epoch	time	train/loss	metrics/accuracy_top1	val/loss	lr/pg0	lr/pg1	lr/pg2
YOLOv12m (seed42_fold 1)	1	60.3206	0.65879	0.47586	0.75684	0.0003	0.0003	0.0003
	5	282.812	0.26333	0.78621	1.22925	0.001443	0.001443	0.001443
	10	561.944	0.23455	1	0.12842	0.00137	0.00137	0.00137
	15	840.813	0.1806	0.98621	0.04895	0.001205	0.001205	0.001205
	20	1119.77	0.18946	1	0.01189	0.00104	0.00104	0.00104
YOLOv12m (seed42_fold 2)	1	61.0958	0.68226	0.47586	0.69653	0.0003	0.0003	0.0003
	5	287.078	0.2499	0.97931	0.11321	0.001443	0.001443	0.001443
	10	568.002	0.24691	0.97241	0.08372	0.00137	0.00137	0.00137
	15	849.137	0.1635	0.77241	0.50793	0.001205	0.001205	0.001205
	20	1130.85	0.15609	0.97931	0.03246	0.00104	0.00104	0.00104
	25	1413.83	0.1369	0.98621	0.1231	0.000875	0.000875	0.000875
	27	1527.68	0.12409	0.97931	0.0274	0.000809	0.000809	0.000809
YOLOv12m (seed42_fold 3)	1	61.2324	0.69578	0.47917	0.68994	0.0003	0.0003	0.0003
	5	286.268	0.27024	0.82639	0.47083	0.001443	0.001443	0.001443
	10	569.906	0.30619	0.77083	0.3114	0.00137	0.00137	0.00137
	15	855.386	0.26075	1	0.00793	0.001205	0.001205	0.001205
	17	968.085	0.23989	1	0.0093	0.001139	0.001139	0.001139
YOLOv12m (seed42_fold 4)	1	61.7307	0.69537	0.52083	0.69604	0.0003	0.0003	0.0003
	5	288.473	0.19366	1	0.02075	0.001443	0.001443	0.001443
	10	575.01	0.22874	0.65278	0.64795	0.00137	0.00137	0.00137
	15	858.476	0.25469	1	0.03178	0.001205	0.001205	0.001205
YOLOv12m (seed42_fold 5)	1	61.5602	0.6864	0.47917	0.69116	0.0003	0.0003	0.0003
	5	288.36	0.19687	0.53472	2.7373	0.001443	0.001443	0.001443
	10	572.199	0.2654	0.92361	0.31	0.00137	0.00137	0.00137
	15	854.126	0.22886	0.95139	0.20398	0.001205	0.001205	0.001205
	17	966.948	0.22133	0.97222	0.05902	0.001139	0.001139	0.001139

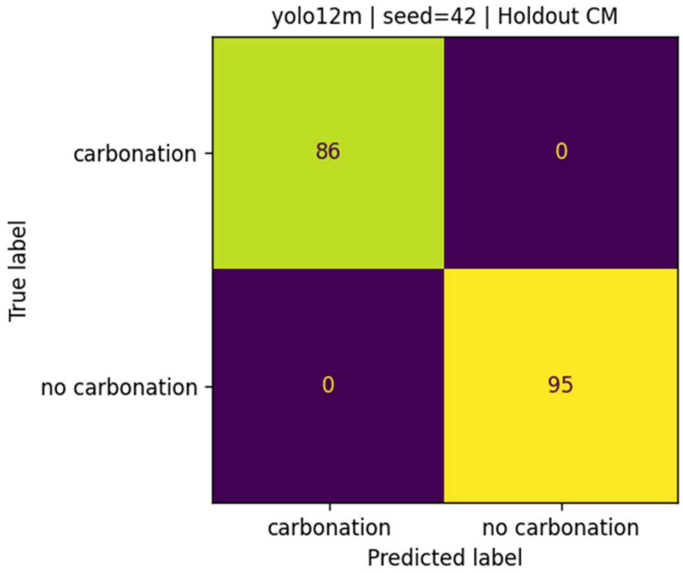

Figure S7. Confusion matrix of the YOLOv12m on the independent hold-out test set (seed=42).

Table S8. Performance metrics values for the YOLOv12m model at seed=123.

	epoch	time	train/loss	metrics/accuracy_top1	val/loss	lr/pg0	lr/pg1	lr/pg2
YOLOv12m (seed123_fold1)	1	61.1167	0.65261	0.47586	0.71143	0.0003	0.0003	0.0003
	5	290.284	0.2807	0.98621	0.03753	0.001443	0.001443	0.001443
	10	573.963	0.33366	0.95172	0.22357	0.00137	0.00137	0.00137
	13	744.008	0.15953	0.97931	0.0903	0.001271	0.001271	0.001271
YOLOv12m (seed123_fold2)	1	61.3089	0.64347	0.47586	0.70996	0.0003	0.0003	0.0003
	5	289.008	0.27812	0.97931	0.53848	0.001443	0.001443	0.001443
	10	572.839	0.2529	0.9931	0.03823	0.00137	0.00137	0.00137
	15	856.868	0.19384	0.97931	0.04853	0.001205	0.001205	0.001205
	20	1140.87	0.17893	0.9931	0.05249	0.00104	0.00104	0.00104
	21	1197.78	0.177	1	0.03679	0.001007	0.001007	0.001007
YOLOv12m (seed123_fold3)	1	61.2452	0.6737	0.47917	0.69165	0.0003	0.0003	0.0003
	5	288.349	0.20055	0.98611	0.03119	0.001443	0.001443	0.001443
	10	572.638	0.2824	0.63194	0.73047	0.00137	0.00137	0.00137
	14	797.417	0.24349	0.57639	2.42383	0.001238	0.001238	0.001238
YOLOv12m (seed123_fold4)	1	61.257	0.6929	0.47917	0.6897	0.0003	0.0003	0.0003
	5	286.539	0.23858	0.95139	0.17535	0.001443	0.001443	0.001443
	10	569.498	0.26361	0.98611	0.07216	0.00137	0.00137	0.00137
	15	850.3	0.18771	0.96528	0.09827	0.001205	0.001205	0.001205
	20	1131.63	0.23126	0.96528	0.12015	0.00104	0.00104	0.00104
	21	1188.14	0.23757	0.99306	0.05267	0.001007	0.001007	0.001007
YOLOv12m (seed123_fold5)	1	61.8246	0.70069	0.47917	0.69141	0.0003	0.0003	0.0003
	5	289.134	0.30047	0.83333	0.66418	0.001443	0.001443	0.001443
	10	571.04	0.29609	0.99306	0.03078	0.00137	0.00137	0.00137
	14	798.184	0.34139	0.86806	0.26471	0.001238	0.001238	0.001238

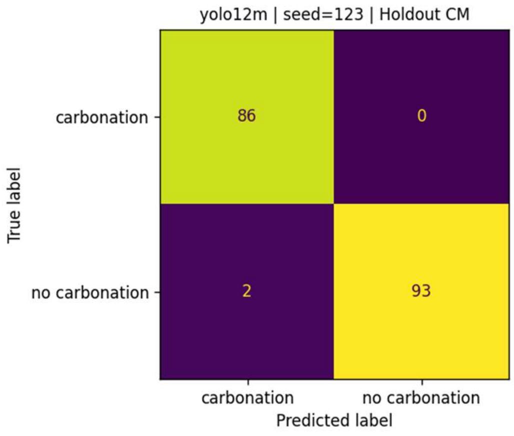

Figure S8. Confusion matrix of the YOLOv12m on the independent hold-out test set (seed=123).

Table S9. Performance metrics values for the YOLOv12m model at seed=456.

	epoch	time	train/loss	metrics/accuracy_top1	val/loss	lr/pg0	lr/pg1	lr/pg2
YOLOv12m (seed456_fold1)	1	60.4865	0.67432	0.52414	0.6897	0.0003	0.0003	0.0003
	5	286.624	0.24473	0.8069	0.37097	0.001443	0.001443	0.001443
	10	565.43	0.18035	0.97241	0.0703	0.00137	0.00137	0.00137
	15	844.07	0.20745	0.97241	0.06053	0.001205	0.001205	0.001205
	20	1124.07	0.14012	0.97931	0.03086	0.00104	0.00104	0.00104
	22	1235.58	0.15587	0.96552	0.12534	0.000974	0.000974	0.000974
YOLOv12m (seed456_fold2)	1	60.6881	0.67463	0.47586	0.72339	0.0003	0.0003	0.0003
	5	283.857	0.23241	1	0.0448	0.001443	0.001443	0.001443
	10	563.692	0.19507	0.9931	0.03101	0.00137	0.00137	0.00137
	15	842.342	0.15154	0.9931	0.02163	0.001205	0.001205	0.001205
YOLOv12m (seed456_fold3)	1	61.0311	0.68475	0.47917	0.69653	0.0003	0.0003	0.0003
	5	283.445	0.31134	0.52083	10.5605	0.001443	0.001443	0.001443
	10	559.781	0.31224	0.98611	0.17973	0.00137	0.00137	0.00137
	15	838.31	0.1955	0.70139	0.49609	0.001205	0.001205	0.001205
	16	894.222	0.19364	0.96528	0.09355	0.001172	0.001172	0.001172
YOLOv12m (seed456_fold4)	1	61.0691	0.69022	0.52083	0.69409	0.0003	0.0003	0.0003
	5	284.07	0.29225	0.90278	0.19174	0.001443	0.001443	0.001443
	10	561.365	0.24641	0.97917	0.04092	0.00137	0.00137	0.00137
	15	840.461	0.26103	0.94444	0.16693	0.001205	0.001205	0.001205
	20	1117.86	0.19048	0.98611	0.05066	0.00104	0.00104	0.00104
	22	1229.29	0.26415	0.99306	0.03298	0.000974	0.000974	0.000974
YOLOv12m (seed456_fold5)	1	61.0206	0.69221	0.47917	0.69092	0.0003	0.0003	0.0003
	5	284.778	0.2673	0.70833	0.60474	0.001443	0.001443	0.001443
	10	562.641	0.28532	0.63889	0.7688	0.00137	0.00137	0.00137
	14	784.099	0.32605	0.96528	0.07446	0.001238	0.001238	0.001238

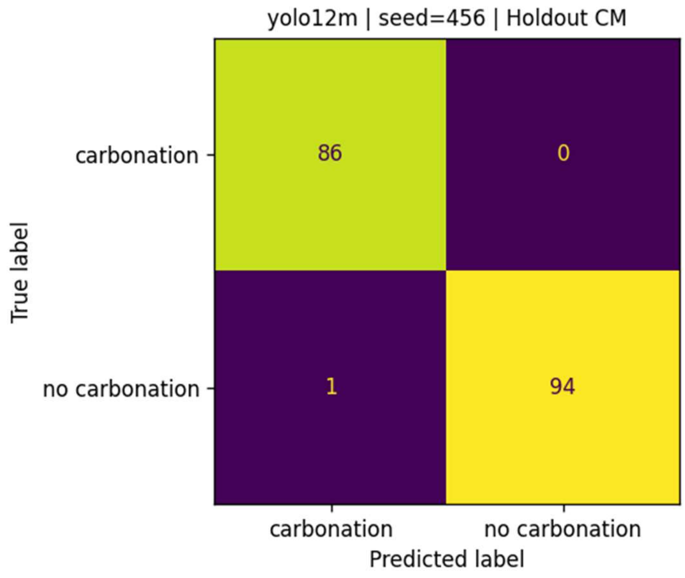

Figure S9. Confusion matrix of the YOLOv12m on the independent hold-out test set (seed=456).

Table S10. Performance metrics values for the YOLOv26m model at seed=42.

	epoch	time	train/loss	metrics/accuracy_top1	val/loss	lr/pg0	lr/pg1	lr/pg2
YOLOv26m (seed42_fold 1)	1	53.6298	0.61704	0.95172	0.42126	0.00015	0.00015	0.00015
	5	268.378	0.26194	1	0.02125	0.000752	0.000752	0.000752
	10	537.371	0.11384	0.68276	7.39453	0.001356	0.001356	0.001356
	15	803.999	0.13088	0.96552	0.07177	0.001205	0.001205	0.001205
YOLOv26m (seed42_fold 2)	1	54.0076	0.61832	0.97241	0.44128	0.00015	0.00015	0.00015
	5	268.32	0.38176	1	0.01162	0.000752	0.000752	0.000752
	10	537.278	0.20509	0.78621	0.6145	0.001356	0.001356	0.001356
	12	643.692	0.5755	0.98621	0.03048	0.001304	0.001304	0.001304
YOLOv26m (seed42_fold 3)	1	53.1144	0.61667	0.98611	0.35938	0.00015	0.00015	0.00015
	5	265.809	0.05283	0.97917	0.07553	0.000752	0.000752	0.000752
	10	532.466	0.34976	0.94444	0.15417	0.001356	0.001356	0.001356
	15	799.338	0.42088	0.98611	0.03413	0.001205	0.001205	0.001205
	19	1014.57	0.14125	0.99306	0.06966	0.001073	0.001073	0.001073
YOLOv26m (seed42_fold 4)	1	53.8016	0.60418	0.93056	0.37634	0.00015	0.00015	0.00015
	5	266.174	0.10758	0.98611	0.13255	0.000752	0.000752	0.000752
	10	533.714	0.36401	0.95139	0.13943	0.001356	0.001356	0.001356
	12	640.879	0.15629	0.95139	0.10736	0.001304	0.001304	0.001304
YOLOv26m (seed42_fold 5)	1	54.0487	0.60904	0.96528	0.37012	0.00015	0.00015	0.00015
	5	267.633	0.06125	0.97917	0.06897	0.000752	0.000752	0.000752
	10	533.188	0.4271	0.22917	4.57617	0.001356	0.001356	0.001356
	12	640.11	0.1999	0.86111	0.8562	0.001304	0.001304	0.001304

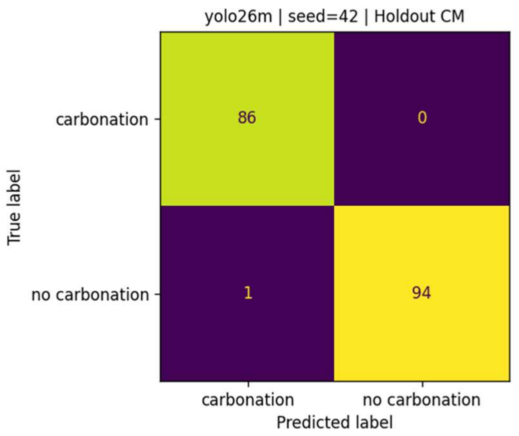

Figure S10. Confusion matrix of the YOLOv26m on the independent hold-out test set (seed=42).

Table S11. Performance metrics values for the YOLOv26m model at seed=123.

	epoch	time	train/loss	metrics/accuracy_top1	val/loss	lr/pg0	lr/pg1	lr/pg2
YOLOv26m (seed123_fold1)	1	53.8022	0.62951	0.95172	0.40173	0.00015	0.00015	0.00015
	5	267.546	0.07535	0.98621	0.0375	0.000752	0.000752	0.000752
	10	535.349	0.2162	0.98621	0.10529	0.001356	0.001356	0.001356
	12	641.972	0.52285	0.9931	0.02173	0.001304	0.001304	0.001304
YOLOv26m (seed123_fold2)	1	54.1551	0.62166	0.91034	0.42639	0.00015	0.00015	0.00015
	5	269.578	0.33419	0.9931	0.01723	0.000752	0.000752	0.000752
	10	539.835	0.23852	0.54483	8.06445	0.001356	0.001356	0.001356
	15	810.202	0.23499	0.64138	1.48926	0.001205	0.001205	0.001205
	17	917.275	0.15868	0.9931	0.05321	0.001139	0.001139	0.001139
YOLOv26m (seed123_fold3)	1	53.6838	0.61602	0.95139	0.37952	0.00015	0.00015	0.00015
	5	266.258	0.06977	0.90972	0.23584	0.000752	0.000752	0.000752
	10	532.6	0.16524	0.79167	0.91919	0.001356	0.001356	0.001356
	12	639.321	0.4883	0.96528	0.40344	0.001304	0.001304	0.001304
YOLOv26m (seed123_fold4)	1	53.9783	0.61985	0.95833	0.39563	0.00015	0.00015	0.00015
	5	269.27	0.04514	0.98611	0.06601	0.000752	0.000752	0.000752
	10	536.239	0.35668	0.95139	0.48621	0.001356	0.001356	0.001356
	15	804.522	0.74225	0.96528	0.10556	0.001205	0.001205	0.001205
	19	1020	0.38054	0.98611	0.05087	0.001073	0.001073	0.001073
YOLOv26m (seed123_fold5)	1	54.3511	0.6168	0.94444	0.40369	0.00015	0.00015	0.00015
	5	269.223	0.0286	1	0.0098	0.000752	0.000752	0.000752
	10	535.948	0.38638	0.77778	0.54858	0.001356	0.001356	0.001356
	12	642.794	0.42216	0.86806	0.63086	0.001304	0.001304	0.001304

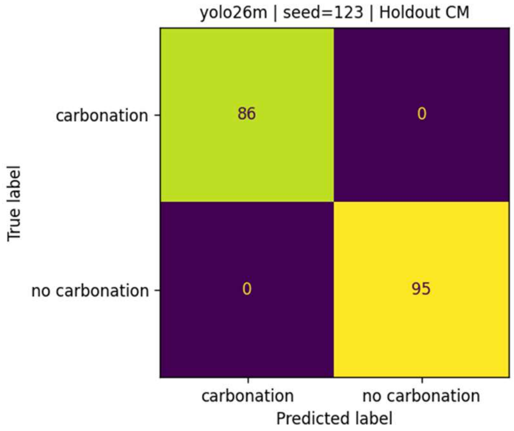

Figure S11. Confusion matrix of the YOLOv26m on the independent hold-out test set (seed=123).

Table S12. Performance metrics values for the YOLOv26m model at seed=456.

	epoch	time	train/loss	metrics/accuracy_top1	val/loss	lr/pg0	lr/pg1	lr/pg2
YOLOv26m (seed456_fold1)	1	53.9553	0.67591	0.86897	0.48572	0.00015	0.00015	0.00015
	5	270.065	0.06847	0.86207	0.28137	0.000752	0.000752	0.000752
	10	538.235	0.18957	0.57931	0.66211	0.001356	0.001356	0.001356
	12	644.402	0.60196	0.92414	0.36993	0.001304	0.001304	0.001304
YOLOv26m (seed456_fold2)	1	53.9224	0.67788	0.82069	0.55151	0.00015	0.00015	0.00015
	5	268.253	0.08253	0.93103	0.13748	0.000752	0.000752	0.000752
	10	535.012	0.271	0.94483	0.24788	0.001356	0.001356	0.001356
	12	640.921	0.53435	0.94483	0.53149	0.001304	0.001304	0.001304
YOLOv26m (seed456_fold3)	1	53.9369	0.66972	0.88194	0.42468	0.00015	0.00015	0.00015
	5	268.589	0.05508	0.97917	0.02659	0.000752	0.000752	0.000752
	10	536.147	0.30919	0.51389	4.43652	0.001356	0.001356	0.001356
	12	642.989	0.26475	0.54167	10.6992	0.001304	0.001304	0.001304
YOLOv26m (seed456_fold4)	1	53.9228	0.67384	0.91667	0.43457	0.00015	0.00015	0.00015
	5	269.92	0.03064	0.95833	0.07298	0.000752	0.000752	0.000752
	10	538.576	0.1841	0.47917	3.86816	0.001356	0.001356	0.001356
	12	646.421	0.52639	0.96528	0.04735	0.001304	0.001304	0.001304
YOLOv26m (seed456_fold5)	1	53.5069	0.66961	0.875	0.50488	0.00015	0.00015	0.00015
	5	268.354	0.05549	0.97222	0.03821	0.000752	0.000752	0.000752
	10	536.271	0.32738	0.89583	0.67295	0.001356	0.001356	0.001356
	15	805.475	0.20304	0.95139	0.14661	0.001205	0.001205	0.001205
	17	913.174	0.53608	0.88889	0.21277	0.001139	0.001139	0.001139

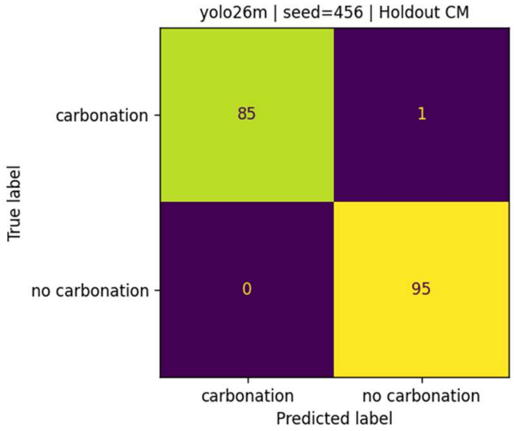

Figure S12. Confusion matrix of the YOLOv26m on the independent hold-out test set (seed=456).
